# Neural Networks or Linguistic Features? - Comparing Different Machine-Learning Approaches for Automated Assessment of Text Quality Traits Among L1- and L2-Learners’ Argumentative Essays

**DOI:** 10.1007/s40593-024-00426-w

**Published:** 2024-09-13

**Authors:** Julian F. Lohmann, Fynn Junge, Jens Möller, Johanna Fleckenstein, Ruth Trüb, Stefan Keller, Thorben Jansen, Andrea Horbach

**Affiliations:** 1 Institute for Psychology of Learning and Instruction, Kiel University, Olshausenstrasse 75, 24118 Kiel, Germany; 2 University of Hildesheim, Hildesheim, Germany; 3 University of Applied Sciences and Arts Northwestern Switzerland, Basel, Switzerland; 4 University of Teacher Education Zürich, Zürich, Switzerland; 5 Leibniz Institute for Science and Mathematics Education at Kiel University, Kiel, Germany; 6 CATALPA, FernUniversität in Hagen, Hagen, Germany

**Keywords:** Automated Essay Scoring, Student Essays, Essay Traits, Feature Engineering, Pre-trained Transformers, Deep-Neural-Networks

## Abstract

Recent investigations in automated essay scoring research imply that hybrid models, which combine feature engineering and the powerful tools of deep neural networks (DNNs), reach state-of-the-art performance. However, most of these findings are from holistic scoring tasks. In the present study, we use a total of four prompts from two different corpora consisting of both L1 and L2 learner essays annotated with trait scores (e.g., content, organization, and language quality). In our main experiments, we compare three variants of trait-specific models using different inputs: (1) models based on 220 linguistic features, (2) models using essay-level contextual embeddings from the distilled version of the pre-trained transformer BERT (DistilBERT), and (3) a hybrid model using both types of features. Results imply that when trait-specific models are trained based on a single resource, the feature-based models slightly outperform the embedding-based models. These differences are most prominent for the organization traits. The hybrid models outperform the single-resource models, indicating that linguistic features and embeddings indeed capture partially different aspects relevant for the assessment of essay traits. To gain more insights into the interplay between both feature types, we run addition and ablation tests for individual feature groups. Trait-specific addition tests across prompts indicate that the embedding-based models can most consistently be enhanced in content assessment when combined with morphological complexity features. Most consistent performance gains in the organization traits are achieved when embeddings are combined with length features, and most consistent performance gains in the assessment of the language traits when combined with lexical complexity, error, and occurrence features. Cross-prompt scoring again reveals slight advantages for the feature-based models.

## Introduction

 Assessing students’ free-text answers (e.g., argumentative essays) is an important task for artificial intelligence (AI) and natural language processing (NLP) in education. This also involves developing tutoring systems based on AI-driven assessment procedures (e.g., Bai & Stede, 2022; Mathias & Bhattacharyya, 2020). Advantages of such systems involve (1) reduced workload for teachers, (2) immediate information about the performance level of their students without extensive manual correction effort, (3) more frequent and instant feedback for students, and (4) a consistent assessment procedure that is, for instance, not bound to human attention processes (see, e.g., Ramesh & Sanampudi, 2022; Uto, 2021; Yan, 2020). Recent research has emphasized the promise of AI-based tutoring systems in supporting students closely during writing and learning processes (e.g., Hussein et al., 2019; Injadat et al., 2021). However, to support students in the context of complex writing tasks such as argumentative essays, an accurate and comprehensive assessment of several aspects of writing is necessary (Bai & Stede, 2022). Such fine-grained scoring of different aspects is a challenging problem that is largely unresolved in the field of automated essay scoring (AES) (see, Horbach et al., 2017; Kumar & Boulanger, 2021). Different AES approaches using machine learning methods have been proposed to face the challenges of AES. Like in many NLP tasks, two general model types have been proposed: feature engineering and deep neural networks (DNN) (Bai & Stede, 2022; Ke & Ng, 2019; Kusuma et al., 2022; Uto, 2021). Recent studies have shown that hybrid models, combining both approaches, can outperform models based on a single resource (e.g., Dasgupta et al., 2018; Uto et al., 2020; see also Mizumoto & Eguchi, 2023). However, most of these comparisons used holistic scoring methods, i.e., assigning one overall grade per essay (Lagakis & Demetriadis, 2021). The holistic approach, however, provides assessment on a superordinate level that is not suitable for meaningful tutorial feedback or in-depth diagnosis of students’ writing abilities (Condon & Elliot, 2022; Narciss, 2008). Moreover, from a methodological point of view, holistic scoring makes it impossible to disentangle possible strengths and weaknesses of the different AES approaches regarding certain aspects of text quality (Andrade, 2018). In the current study, we therefore compare the performance of different AES approaches scoring analytic essay rubrics (also referred to as *traits* in the following). For this purpose, we use four different argumentative prompts, containing English essays written by L1 students (from the ASAP corpus^1^) and L2 students (from the MEWS corpus, Keller et al., 2020; Rupp et al., 2019). We consider analytic benchmark scores assigned by trained human raters representing different aspects of text quality, such as *language quality*, *organization*, and *content*. In doing so, we compare the two single-input approaches to analytic scoring (linguistic features vs. essay-level contextual embeddings from DistilBERT) and explore which approach is superior regarding a given aspect of text quality. First studies comparing these approaches for specific text characteristics, such as lexical complexity, indeed imply performance differences (e.g., Crossley & Holmes, 2023). Furthermore, we investigate whether a hybrid model indeed outperforms the two single-resource approaches across prompts and traits. In addition, we use addition and ablation tests (i.e., stepwise removal/addition of certain groups of features) to uncover types of linguistic features that are especially important for specific traits and that can hardly be captured by (essay-level) contextual embeddings and making them particularly relevant for the hybrid architecture. Finally, we examine the cross-prompt performance of the models within the L1 and L2 corpora. We aim to answer the following research questions (RQ) that guide our experiments:

1. How do models based on linguistic features and text-level contextual-embedding-based models differ regarding their performance on scoring certain aspects of text quality?
2. Under which conditions does a hybrid approach outperform the single-resource models across different aspects of text quality?
3. Which linguistic feature types carry information not covered by the contextual embeddings of DistilBERT and are therefore most important for the hybrid approach?
4. How do the different model architectures differ regarding cross-prompt performance?

The remainder of this article is organized as follows. Section “Overview of Different AES Approaches” introduces different AES approaches and discusses their respective presumed advantages and disadvantages. Section “Method” describes the datasets, the different model architectures, and the training procedures used in the present study. In Section “Results”, we present the results of our experiments. Finally, we discuss our results and outline limitations along possible directions for future research in Section “Discussion”.

## Overview of Different AES Approaches

Most machine learning (ML) approaches to AES follow a supervised learning strategy (Ke & Ng, 2019), where humans’ assessments of a given set of student essays are used as benchmark scores to train ML models. The trained models are then used to assign scores to new texts written in response to the same or new prompts.

Key characteristics of ML models used in supervised learning AES tasks are (1) the way texts (i.e. student essays) are represented as numerical input vectors (i.e. features) and (2) the actual ML architecture being employed (e.g., linear regression or DNN; see Ramesh & Sanampudi, 2022). Both aspects are interrelated, which has led to two overarching AES approaches being established in previous research: feature engineering and DNN approaches (Ke & Ng, 2019; Kusuma et al., 2022). In the feature engineering approach, domain experts select and manually design the text features to be used for a specific task in step (1), combining these hand-crafted linguistic features with predominantly shallow learning techniques in step (2). Conversely, DNN approaches autonomously learn a suitable representation of the features in step (1) and subsequently (or simultaneously) learns a DNN to score essays in step (2). In the upcoming section, we will outline key characteristics of both approaches. Our focus will be on different model inputs most relevant for the comparisons carried out in the present study (see Bai & Stede, 2022; Uto, 2021). A comprehensive overview of AES approaches can be found in Ramesh and Sanampudi (2022), Lagakis and Demetriadis (2021), and Uto (2021).

### Feature Engineering Approach

AES models following the feature engineering approach are based on a theory-driven way of translating text into numerical data using NLP methods (Chen & Meurers, 2016; Crossley, 2020; McNamara et al., 2014; Zesch et al., 2015). Those features range from simple length-based representations, such as the number of words or paragraphs, to highly elaborated linguistic constructs, such as coherence (Mesgar & Strube, 2018) or cohesion measures (e.g., Crossley et al., 2017).

The feature engineering approach is the traditional AES approach (Lagakis & Demetriadis, 2021). Over the last decade, many tools have been proposed that provide the user with a vast range of linguistic features (Chen & Meurers, 2016; Crossley, 2019; Kumar & Boulanger, 2021; Kyle et al., 2018). In the following sections, we will provide a brief overview of common types of features applied in AES tasks.

#### Length and Occurrence Features

In the context of formal education, student essays are written under a specific time limit (*time writing*, see, Weigle, 2002). Therefore, essay length in terms of words, sentences, or paragraphs, has been demonstrated to be a powerful predictor of human scores (see, e.g., Fleckenstein et al., 2020; Zesch et al., 2015). Furthermore, ratios of words per sentence or sentences per paragraph can be interpreted as a proxy for syntactic complexity (Crowhurst, 1983). In addition, many traditional readability metrics consider word and sentence length (Pitler & Nenkova, 2008). Other length features typically used in AES models are mean word length (in characters) or the total number of unique words (e.g., Chen et al., 2016).

Occurrence features are closely related to length-based features and include, for instance, the counting of instances of certain part of speech classes, such as nouns, proper nouns, verbs, adjectives, as well as special characters. Classification of words into word types is known as part-of-speech tagging (POS; e.g., Mitkov & Voutilainen, 2012). In addition, the ratios of specific word types to the total number of words are also frequently used (X. Chen & Meurers, 2016).

#### Error Features

Another important aspect of student writing that usually factors into performance evaluations is the number of errors (e.g., typos or grammar errors). Thereby, error ratios are often calculated, such as the proportion of spelling errors to the total number of words. LanguageTool^2^ is a powerful tool to automatize error counts.

#### Features Relating to Lexical Diversity and Sophistication

A common indicator for the lexical diversity of student essays is type-token ratio (e.g., Richards, 1987). *Tokens* are defined as all individual words in a text whereas *types* are defined as unique words. Thus, if the type-token ratio is close to one, lexical diversity is high. If the type-token ratio is close to zero, lexical diversity is low.

Common features to represent the lexical sophistication of essays are (weighted) counting of occurrences on large word-frequency corpora such as the British National Corpus (BNC) or the Brown frequency list. For example, to determine the predominant use of “easy words”, the top 1000 of the BNC word list have been used as a reference (X. Chen & Meurers, 2016). Conversely, “difficult words”, for example, have been defined as not being included in the top 2000 of the BNC word list (X. Chen & Meurers, 2016). However, these values are rather arbitrary and might also be adapted and aligned with the respective students’ characteristics.

#### Morphological Complexity Features

Morphological complexity measures are related to type-token ratio, but instead of reflecting lexical diversity, they capture the range of different inflections used (Brezina & Pallotti, 2019). For instance, a text with diverse inflected forms such as “writing, wrote, writes” would be deemed to have a higher morphological complexity than one that merely repeats the same form like “writing, writing, writing”. Morphological complexity measures can be calculated by taking the ratio of unique inflectional forms to the sum of all tokens of a given word class (per text, paragraph, or sentence), offering a quantitative insight into the diversity of morphological forms (Brezina & Pallotti, 2019).

#### Syntactic Complexity Features

*Dependency parsing* captures the grammatical relationships between words, offering a structured representation of sentences that reveals their underlying syntactic properties (e.g., Nivre, 2010). Dependency parsing has been used in AES context to count, for instance, the number of fragment clauses, prepositional phrases, coordinate clauses, or relative clauses (e.g., X. Chen & Meurers, 2016). This can provide valuable insight into a student’s ability to compose sophisticated sentences and present complex topics and ideas.

#### Cohesion Features

Cohesion refers to the lexical linking within a text, providing the reader with a sense of flow and consistency (Crossley et al., 2016). In general, more cohesive texts allow the reader to better follow the ideas presented and to understand links between different topics (Crossley et al., 2017). The lexical overlap between consecutive text segments, such as sentences or paragraphs, can numerically operationalize cohesion. Another measure of cohesion is, for instance, the frequent usage of connectors that structure the essay.

#### Feature Engineering Approach – Advantages and Challenges

One of the main advantages of the feature engineering approach is the theory-driven way of pre-processing the text-inherent information before feeding it into an ML model. This process of creating features provides a high degree of control over what information may be used by the algorithm. Feature engineering and feature selection might also be adapted to specific types of essays or learner populations. Furthermore, the explicit, theory-based approach of feature selection forms a prerequisite for explainable AES scores (answering how a given score is determined). Therefore, the feature-based approach has usually been combined with ML model architectures that allow a high amount of explainability, such as linear regression, logistic regression, random forests, or decision trees. This approach, however, is rarely combined with DNNs, whose hidden layers (often referred to as the “black box” of DNNs) make it difficult to understand and interpret the calculation of scores from a subjective point of view.

One primary challenge of the feature-based approach is the adequate representation of content, an element many consider pivotal, if not the most critical aspect of essays (e.g., Ramesh & Sanampudi, 2022; see also Perelman, 2014). A potential strategy to incorporate content in the realm of feature engineering without a loss of explainability is the application of bag-of-words or n-gram techniques (e.g., Ke & Ng, 2019). These methods employ either word frequencies (uni-grams) or word sequence frequencies (n-grams) to represent an essay’s content in AES tasks. Typically, these approaches are used in conjunction with stop-word filtering and lemmatization. Nevertheless, the representation of content through bag-of-words or n-gram techniques remains significantly limited, reducing it merely to the occurrence of specific words or chunks. This fails to account for the contextual nature of language, wherein a word’s meaning heavily relies on its surrounding lexical environment. Additionally, n-gram techniques pose a risk of feature explosion when every word and word sequence within a given text corpus is represented as an independent feature. However, a powerful alternative to process text data and encode the content of a text has been proposed in the context of DNNs, namely word embeddings.

### Deep-Neural-Networks

Recent applications of DNNs in AES primarily rely on word embeddings (e.g., Beseiso & Alzahrani, 2020; Rodriguez et al., 2019; Uto et al., 2020). The basic idea of word embeddings is to represent the meaning of words with specific loadings (i.e., numerical values) on several latent dimensions. Each dimension represents a different (and largely unknown) aspect of semantic meaning. Each word has a unique set of loadings representing its meaning as a vector in an *N*-dimensional semantic space. Words with similar meanings have similar loading patterns (i.e., a similar vector representation in the semantic space). The number of latent dimensions *N* serves as a hyperparameter and can be set to arbitrary values. For instance, the embedding layer of the BERT-base model consists of 768 dimensions. Training embedding models involves a DNN that learns to predict words based on their surrounding words (i.e., the context). After extensive training on large samples of authentic texts, the final embeddings capture nuanced semantic relationships, such as syntactic and thematic similarity between words. Currently, pre-trained vector spaces, such as Word2Vec, are accessible and have been trained on extensive text corpora.

Based on such word embeddings, several text-processing DNN architectures, like recurrent-neural networks (RNNs) or long-short-term models (LSTMs), have been developed, and many of them have also been adopted for AES tasks (e.g., Alikaniotis et al., 2016; Taghipour & Ng, 2016; Uto & Okano, 2020). One further challenge in processing text arises from the fact that the meaning of a word is never fixed, but highly affected by the context in which it appears. Thus, the words’ latent representations should not be fixed either, but rather changed and adapted according to context. To tackle this issue, various advanced model architectures have been proposed, with attention mechanisms representing a groundbreaking development (Vaswani et al., 2017). Attention mechanisms facilitate the dynamic adjustment of word embeddings based on the surrounding words, enabling models to better capture the meaning of words in a given context. The implementation of such attention mechanisms in large pre-trained transformer models has recently led to significant improvements and breakthroughs in various NLP tasks, such as text classification (e.g., Yang et al., 2019), translation (e.g., Lample & Conneau, 2019), or summeriazation (e.g., Lewis et al., 2019).

In AES, the application of transformer models has improved state-of-the-art performances (Bai & Stede, 2022; Uto et al., 2020; Wang et al., 2022; Xue et al., 2021). In text classification or regression tasks using transformers like BERT (i.e. encoder models), a fixed-length text-level representation is required that is independent of the number of words or tokens in a given text. Consequently, researchers often employ text-level pooling methods (Shen et al., 2018) or utilize a special token representation, such as the CLS token approach (e.g., Uto et al., 2020). These strategies provide a contextualized representation of the text with a consistent length, which can be effectively used as features for the regression or classification tasks at hand (Mayfield & Black, 2020).

On the one hand, DNNs provide a powerful approach to AES with no need for elaborated feature engineering and with the promise of capturing content much better than n-gram or other content feature approaches such as prompt-similarity analysis or topic dictionaries. On the other hand, contextual embeddings are latent representations of textual information, which complicates the goal of explainable AES.

### Hybrid Models

Several recent AES applications have suggested that contextual embedding-based DNNs and feature engineering approaches should not be considered as competing (see, e.g., Ke & Ng, 2019), but rather as complementing each other by forming a combined model (Bai & Stede, 2022; Kusuma et al., 2022). As demonstrated, for instance, by Uto et al. (2020) or Beseiso and Alzahrani (2020), such combined models, typically referred to as *hybrid models*, can outperform single-resource models (see also, e.g., Dasgupta et al., 2018 and Mizumoto & Eguchi, 2023). This result seems quite intuitive, as both approaches use different strategies to process text data and thus might capture different aspects of essay quality. However, the application of hybrid models has so far only been applied to holistic scoring. Using holistic scoring tasks makes it impossible to determine which approach has its merits in terms of which aspects of text quality. This limitation might be overcome with analytic AES applications.

## Method

To address our research questions, we analyzed argumentative student essays written in response to different argumentative writing prompts. Using only argumentative prompts ensured that similar aspects of text quality (also referred to as *traits* in the following), were relevant across prompts and corpora. Additionally, the aspect of content is generally of particular importance in argumentative essays. We used English essays from L1 and L2 learners to assess the generalizability of the results across different learner populations (see, e.g., Crossley, 2020).

### Datasets

To compare the performance of different AES approaches regarding different aspects of text quality, we used four argumentative prompts from two different corpora. Two of these prompts ($\:{N}_{1}=1783;{N}_{2}=1800)\:$stem from the widely-used ASAP competition. These two prompts are the only ones from the ASAP corpus that involve argumentative writing. Both prompts contain essays written by US-American L1 learners. Mathias and Bhattacharyya (2018) introduced analytic labels for these two prompts via the so-called ASAP + + annotation, covering five aspects of text quality: *content*, *organization*,* word choice*,* sentence fluency*, and *conventions* (more details can be found in the Appendix and in Mathias & Bhattacharyya, 2018).

To expand our analyses to L2 learners, we also included two prompts ($\:{N}_{3}=1179;{N}_{4}=1112)\:$from the MEWS corpus (*Measuring Writing Skills in English as a Second Language*; Fleckenstein et al., 2020; Rupp et al., 2019). The MEWS corpus contains argumentative essays written by German and Swiss L2 upper secondary school students (Keller et al., 2020). The two prompts required students to write essays on the following topics: (1) whether advertising to young children should be allowed (AD prompt) and (2) whether it is more important for teachers to possess excellent content knowledge or to relate well with students (TE prompt). The essays were labeled analytically in the context of the TrACE project (Training Assessment Competencies in English as a Foreign Language; Keller et al., 2024). The analytic labels contain three traits: *content*, *organization*, and *language quality*. This dataset is available on OSF^3^.

**Fig. 1:**
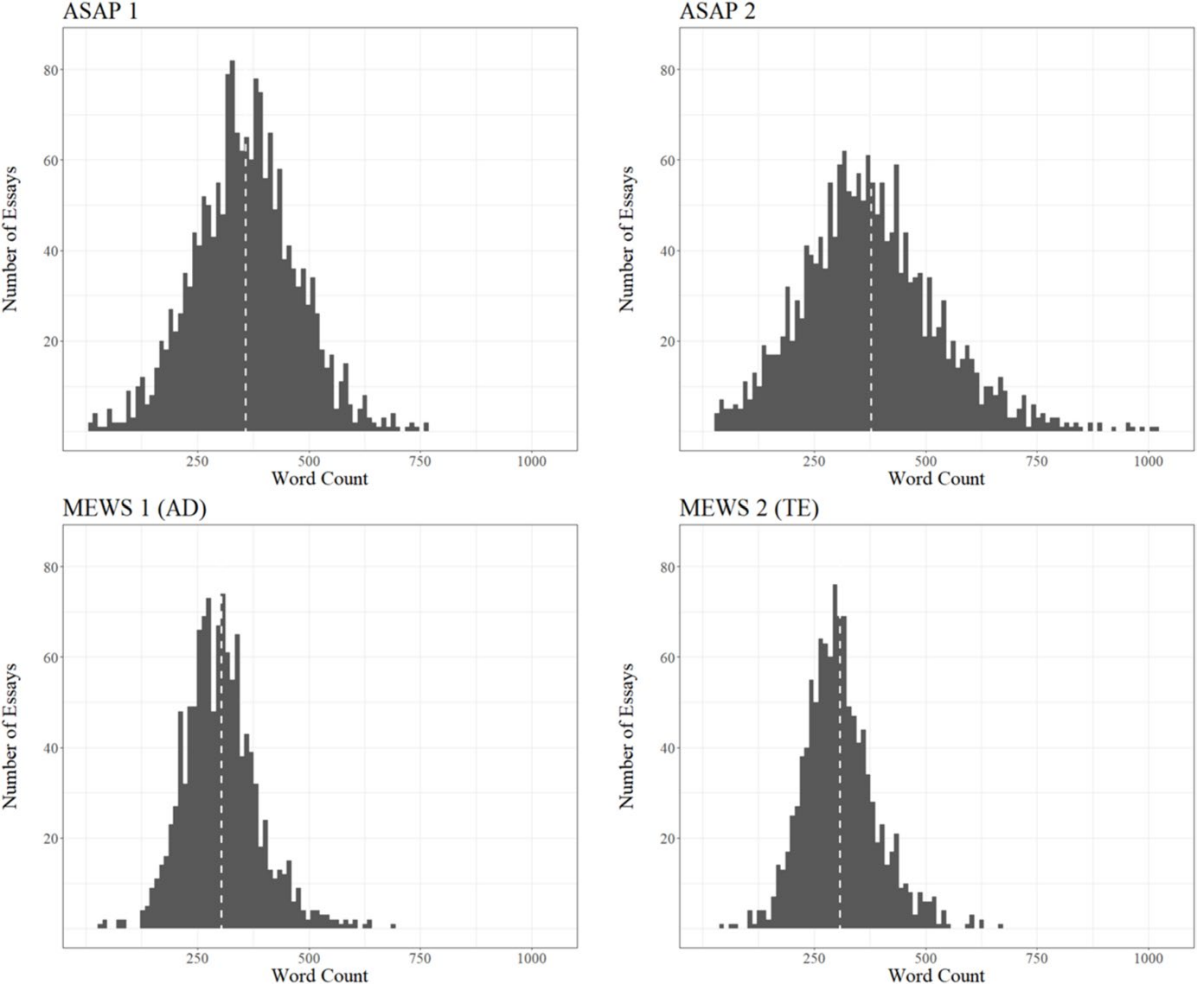
Distributions of Essay Length Counted in Words. The white dashed lines mark the respective mean values

The four writing prompts, as well as further information and descriptive statistics, can be found in Table 1. Figure 1 represents the prompt-specific distributions of essay lengths. The essays of the L1 learners are slightly longer on average and the distribution is noticeably wider.

**Table 1:** Prompts of ASAP and MEWS used in the Present Study

Corpus	Learners	Number of labelled essays	Mean essay length in tokens (SD)	Essay traits	Score range	Prompt
ASAP	L1	1783	357.3 (115.7)	Content Organization Word Choice Sentence Fluency Conventions	1–6	More and more people use computers, but not everyone agrees that this benefits society. Those who support advances in technology believe that computers have a positive effect on people. They teach hand-eye coordination, give people the ability to learn about faraway places and people, and even allow people to talk online with other people. Others have different ideas. Some experts are concerned that people are spending too much time on their computers and less time exercising, enjoying nature, and interacting with family and friends. Write a letter to the editor of a newspaper about how computers affect society today.
ASAP	L1	1800	377.4 (153.5)	Content Organization Word Choice Sentence Fluency Conventions	1–6	Write a persuasive essay to a newspaper reflecting your views on censorship in libraries. Do you believe that certain materials, such as books, music, movies, magazines, etc., should be removed from the shelves if they are found offensive? Support your position with convincing arguments from your own experience, observations, and/or reading.
MEWS (AD)	L2	1179	303.4 (84.0)	Content Organization Language	0–6	Do you agree or disagree to the following statement: Television advertising directed toward young children (aged two to five) should not be allowed.
MEWS (TE)	L2	1112	308.0 (82.1)	Content Organization Language	0–6	Do you agree or disagree to the following statement: A teacher’s ability to relate well with students is more important than excellent knowledge of the subject being taught.

#### Benchmark Scores and Rater Effects

While the ASAP + + trait scores are already provided as adjudicated true scores, the TrACE trait scores were available as double-rated raw rater data (i.e., at least two scores per essay and analytic rubric; details can be found in Appendix Table 7 and in Keller et al., 2024). As proposed by Uto and Okano (2020), we employed an IRT-based rater model to account for systematic rater effects. To do so, we used the software *Facets* (Linacre, 2019). However, to keep model complexity low, we decided to account for rater severity effects only (not, for instance, for rater centrality/extremity or consistency, but see Uto & Okano, 2020 or Robitzsch & Steinfeld, 2018, for alternative rater modeling approaches, which can also be combined with AES models). The derived essay scores after controlling for rater effects, are on a continuous scale but limited to the original scoring range (Linacre, 1994).

**Fig. 2:**
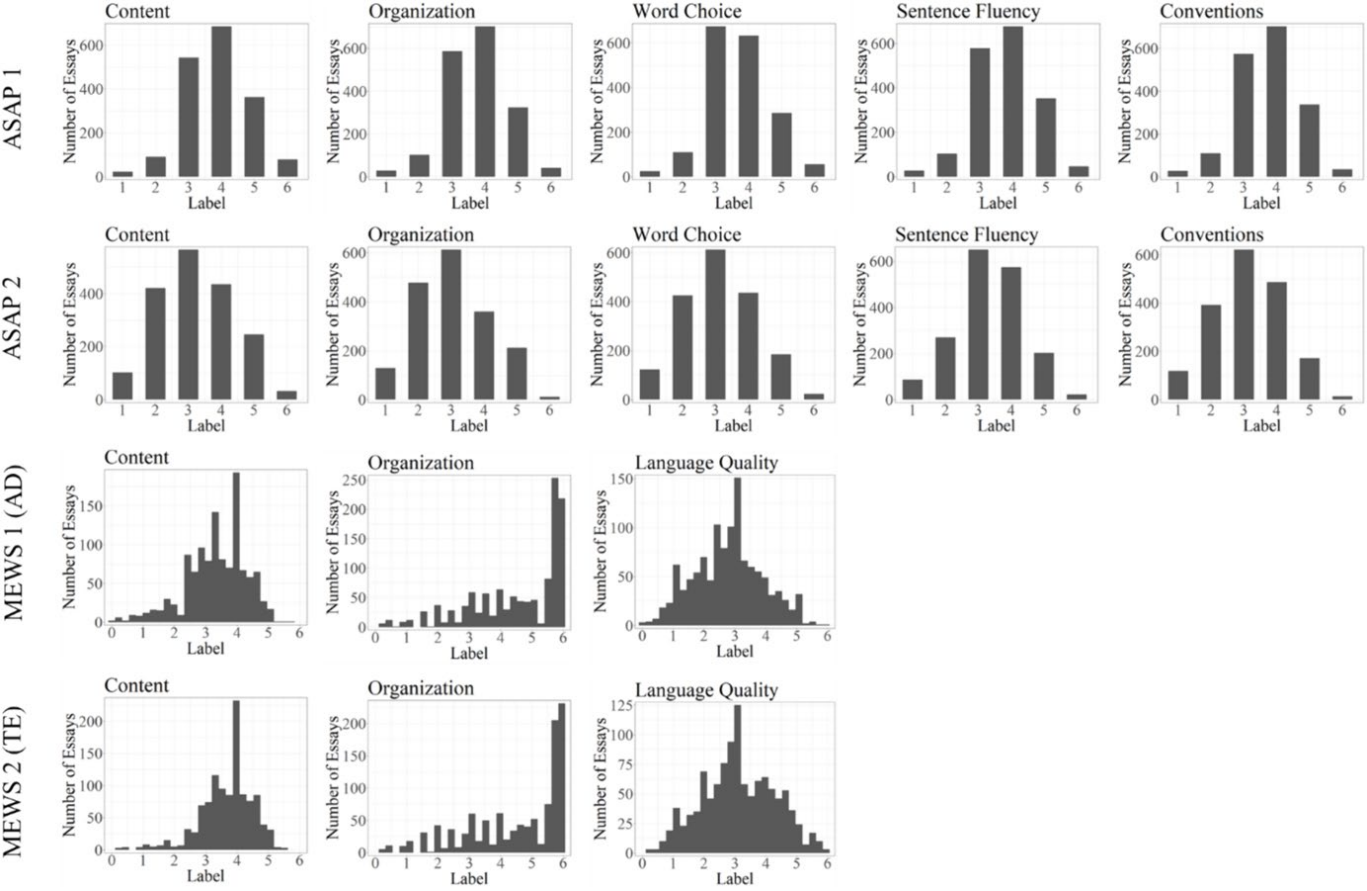
Distributions of Essay Trait Labels

Figure 2 shows the distributions of the analytic target labels of each essay corpus. All targets are approximately normally distributed, except for the two organization traits of MEWS 1 and 2, which are highly negatively skewed.

### Model Inputs

**Fig. 3:**
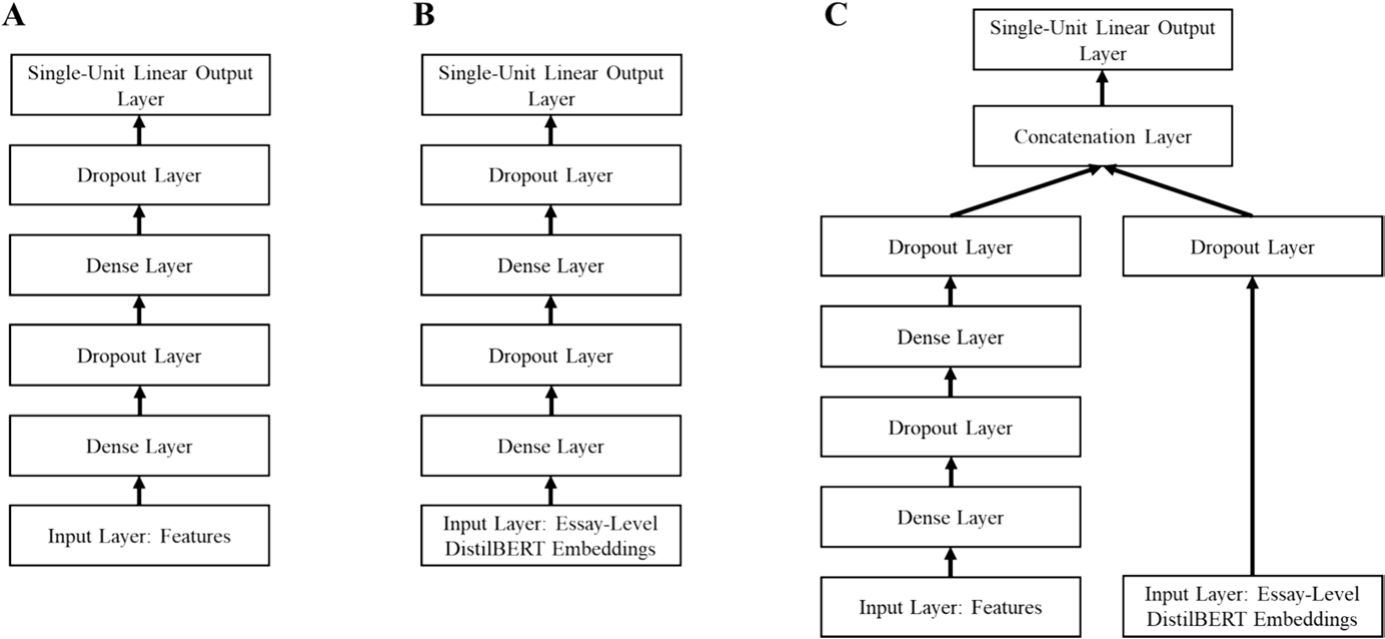
Feature-Based (**A**), Contextual Embedding-Based (**B**), and Hybrid (**C**) DNN Architectures. Number of layers, dropout rate, and number of units per Dense layer were varied during the random search procedure

Our guiding research questions focus on the performance of different ML approaches to AES that rely on different input resources. We created a standard DNN architecture (Fig. 3A and B) in tensorflow (TensorFlow Developers, 2024), which was used for all input types. However, it is well-known that model performance depends not only on the characteristics of the input vectors (e.g., linguistic features vs. contextual embeddings vs. hybrid) but also on the model architecture (e.g., depths of the DNN) and the fit between the input vector and the model architecture. We, therefore, systematically varied the hyperparameters of the model architecture in a random search procure (e.g., Bergstra, & Bengio, 2012). This procedure is described in detail in the subsection *Training procedures*. In the following, we first introduce the two different types of model inputs – linguistic features and contextual embeddings.

#### Linguistic Features

We created a set of 220 different linguistic features representing relevant text features typically included in feature-based AES models following X. Chen & Meurers, 2016; Ke & Ng, 2019; Kumar & Boulanger, 2021; Zesch et al., 2015. Table 2 presents all feature types with examples from our feature set (a comprehensive list of all 220 features can be found in the Appendix Table 8 and on OSF^4^).

Automatic scoring of student essays benefits from a comprehensive analysis of different types of linguistic features. Previous studies suggest that these features constitute relevant dimensions of text quality that can differentiate students’ writing abilities (Attali & Powers, 2008; Deane et al., 2024). For example, length features provide a surface measure of the extensiveness of an essay and its elements, such as sentences and paragraphs, and thus reflect the student’s ability to develop their ideas within a certain amount of time (Shermis & Burstein, 2003). Occurrence features, which count the frequency of specific words or structures, help identify key elements and themes within the text (e.g., Chassab et al., 2021). Error features are crucial for assessing the correctness of language use, highlighting issues in grammar, spelling, and punctuation (e.g., Gamon et al., 2013). Morphological complexity features examine the use of different word forms and structures, indicating the student’s command over language intricacies (Gamon et al., 2013). Cohesion features measure how well the essay’s parts fit together, revealing the student’s ability to create a coherent and logically flowing argument (e.g., Crossley et al., 2016). Readability features assess how easily the text can be understood, which is essential for effective communication (Pitler & Nenkova, 2008). Lexical diversity features indicate the range of vocabulary used, showcasing the student’s linguistic richness and variation (e.g., Jarvis, 2013). Lexical sophistication features highlight the use of advanced and nuanced vocabulary, reflecting a higher level of language proficiency (Crossley, 2020). Lastly, syntactic complexity features analyze the structure of sentences, showing the student’s skill in constructing varied and complex sentences, which is a hallmark of advanced writing ability (Crossley & McNamara, 2014). Together, these features provide a multidimensional assessment of essay quality, capturing both surface-level and deeper linguistic competencies. Empirical studies show that these features types can be relevant in AES tasks (e.g., Crossley, 2020).

We used the Python library spaCy^5^ for POS tagging and dependency parsing, LanguageTool^6^ for error detection, and the BNC, SUBLEX, and NGSL word lists as well as word lists from the Psycholinguistic Database (e.g., brown frequency list) to count easy words (i.e., frequently used words in large text corpora) and difficult words (i.e., less frequently used words in large text corpora).

#### DistilBERT’s Contextual Embeddings

Recent comparisons and reviews of AES applications employing pre-trained transformer models indicated that performance hardly increases when these models are fine-tuned (Mayfield & Black, 2020; Rodriguez et al., 2019). Additionally, runtime and computational demands largely increase due to the extensive fine-tuning processes when using such large models. To keep runtime low, we followed suggestions by Mayfield and Black (2020) and used the distilled version of BERT (DistilBERT; Sanh et al., 2019).

In our present experiments, we focus on the question of how contextual embeddings perform in AES for different traits (e.g., Beseiso & Alzahrani, 2020; Mayfield & Black, 2020; Nadeem et al., 2019). Therefore, we did not fine-tune DistilBERT and kept all its layers frozen (i.e., not trainable) during our experiments. However, we supplemented these non-trainable DistilBERT layers with an essay-level maximum pooling layer (e.g., Shen et al., 2018) and additional Dense layers. In doing so, we received a contextual embedding vector of length 768 from DistilBERT for each essay, which served as the input vector for our AES architecture (Fig. 3B).

### Training Procedure and Model Architectures

#### Main Experiments

To train and evaluate our models, we followed a five-fold cross-validation strategy. For the ASAP datasets, we employed the splits introduced by Taghipour and Ng (2016) that imply five 60-20-20 splits in training, validation, and test data. As the test dataset of the ASAP competition is not publicly accessible, the experiments are based solely on the training data of the ASAP competition (see, Taghipour & Ng, 2016)^7^. These predefined splits had also been used for trait scoring of the ASAP + + dataset by Mathias and Bhattacharyya (2018, 2020).

For the MEWS corpus, we also employed five-fold cross-validation. However, because of the considerably smaller datasets in MEWS, we decided to use 70% of each dataset as the training set, 10% of the data as the validation set, and 20% as the test data in each fold. To find the best epoch for each run, we used an early-stopping callback function that tracked the validation loss. The model showing the best performance on the validation set across folds was finally used for evaluation with the test data.

All DNN model architectures were set up in tensorflow (python code can be found on the OSF repository^8^). We designed our AES models as regression models. The values indicating the trait-specific essay qualities are ordinally scaled. Since they range, for instance, from 1 = *high quality* to 6 = *low quality* and thus can be assumed to be continuous, we decided against classification approaches (see Beseiso & Alzahrani, 2020, for a comparison of classification vs. regression AES models). Thus, we used a single unit with linear activation in the output layer and the mean squared error (MSE) as the loss function in all DNN architectures.

**Table 2:** Feature Types with Examples from the Feature Set

Feature type	Number of features	Example features
Length features	15	Number of words Number of paragraphs Number of sentences
Occurrence features	30	Number of nouns Number of formal words Number of unique nouns
Error features	9	Error ratio Grammar error ratio Punctuation error ratio
Morphological complexity features	11	Number of finite verbs Number of non-third person singular verb Ratio of comparatives
Cohesion features	8	Number of connectors Number of unique connectors Mean noun overlap with previous sentence
Readability features	15	Flesch score Integration cost Average number of sentences per 100 words
Lexical diversity features	59	Type-token ratio Type-token ratio lexical words Global edit distance
Lexical sophistication features	62	BNC easy word ratio SUBLEX easy word ratio Brown Frequencies lexical word ratio
Syntactic complexity features	6	Number of subordinate clauses Number of fragment sentences Mean tokens before main verb

We employed the Adam optimizer and the Mean Squared Error (MSE) loss function. For each trait of each prompt, different models were trained varying the type of input (features vs. embeddings vs. hybrid). In addition, we systematically changed the hyperparameters defining the model architectures to ensure valid comparisons across the different AES approaches. In doing so, we used a random search procedure (Bergstra & Bergio, 2012) varying the following hyperparameters: learning rate (5e^−3^, 1e^−3^, 5e^−4^, 1e^−4^), number of dense layers (0^9^, 1, 2) units per dense layer (64, 128, 256) and dropout rates (0.2, 0.3, 0.4, 0.5, 0.6). During the random search, we tested 50% of this parameter space.

#### Hybrid Model

The hybrid architecture used both input resources – linguistic features and essay-level contextual embeddings from DistilBERT. In the first step, the two types of inputs were separately processed through additional Dense and Dropout layers in two parallel model parts (Fig. 3C). The hyperparameters of the feature input part were determined by the best performing models from the corresponding feature-based models. However, it turned out that the hybrid architecture was much more difficult to optimize and that additional Dense Layers hardly improved model performance of the embedding-based models (see Appendix Table 9). Therefore, we decided to employ a reduced second parallel model part for the embeddings. This second part using the embedding input was only fed through one additional dropout layer and then directly into the concatenation layer (Fig. 3C). As the first part of the model architecture was fixed, we only varied the dropout rate for the second (i.e., the embedding input) part of the model (0.3, 0.4, 0.5, 0.6, 0.7) and the learning rate of the Adam optimizer (5e^−3^, 1e^−3^, 5e^−4^, 1e^−4^). The concatenation layer was incorporated as the last stage of the hybrid model before the (single-unit) output layer. This implies that interactions between linguistic features and contextual embeddings were enabled in this hybrid architecture (Fig. 3C).

#### Addition and Ablation Tests

RQ 3 was concerned with the question which linguistic feature types were not covered by the contextual embeddings. To answer RQ 3, we ran two types of tests to gain more insights into the interplay of contextual embeddings and linguistic features. In the first series of tests (addition), we always started with the embedding-based DNN. From there on, we ran a reduced form of the hybrid model, supplementing the contextual embeddings with one feature group at a time. In doing so, we distinguished nine types of features (see Table 2). Thus, we reran each trait- and prompt-specific model nine times using the same cross-validation procedure as in the main experiment.

For the second series of tests (ablation), we took the full hybrid model as the starting point. In an iterative process, we reran each model nine times, each time removing a different feature group from the input. Again, we applied the same cross-validation procedure as in the main experiments.

#### Cross-Prompt Scoring

For cross-prompt scoring (RQ 4), we relied on the hyperparameter settings of the respective best-performing model of each prompt and trait. Again, we employed the cross-validation procedure outlined above but used the complete data from the respective other prompt within a given corpus as the test data instead.

#### Two Linear Regression Baselines

We additionally compared the three DNNs to two simpler baseline models from Scikit-learn^10^. In doing so, we (1) combined a linear ridge regression with an N-gram-vectorizer with stop word filtering as input and (2) a linear ridge regression with our feature set as input (which will be described in the following sub-section). The n-gram baseline models allowed us to disentangle whether and to what extent more complex text processing, as provided by pre-trained transformer models using embeddings and attention mechanisms, are superior to simpler text processing using the classical n-gram approach in automated essay trait scoring tasks. Furthermore, the feature baseline model allowed us to explore whether (and to what extent) complex model architectures (i.e., DNN architectures) are superior compared to simple linear models in automated essay trait scoring. To fit the baseline models, we used the same cross-validation procedure and a grid search approach varying n-gram range (unigrams, bigrams, trigrams) and the alpha parameter of the ridge regression (1e^−4^, 1e^−3^, 1e^−2^, 1e^−1^, 1, 10, 100). The best-performing model on the validation sets across folds was evaluated with the test data.

### Evaluation Metrics

We used quadratic weighted kappa (QWK; Cohen, 1968) as evaluation metric. QWK is the most frequently used metric in AES tasks and had also been reported in the course of previous analyses of the ASAP + + datasets (Mathias & Bhattacharyya, 2018, 2020).

A QWK value of one indicates perfect agreement between predicted scores and benchmarks, a value of zero corresponds to a chance agreement and a negative value represents systematic disagreement, with minus one as the extremum corresponding to complete disagreement.

To also compare model- and trait-specific performance across prompts, we employed average QWK ($\:\stackrel{-}{\text{Q}\text{W}\text{K}}$; see, e.g., Taghipour & Ng, 2016) as well as mean QWK differences ($\:\stackrel{-}{\varDelta\:\text{Q}\text{W}\text{K}}$). However, as averaging QWK across different scales is notoriously problematic (e.g., Doewes et al., 2023), we also used average Pearson Correlation Coefficient (PCC) as an additional metric to compare model performance across traits.

#### T-Tests

To examine whether one approach (feature vs. embedding vs. hybrid) performed significantly better than the other approaches, we used pairwise T-tests (see, e.g., Uto et al., 2020). We employed one-sided testing for comparisons against the baseline models.

## Results

Tables 3 and 4 present the trait-specific test data performances in QWK after the random search cross-validations of our main experiments for ASAP and MEWS, respectively. The best-performing hyperparameter settings for each model can be found in the Appendix (Table 9).

### Features Versus Contextual Embeddings

In RQ 1, we aimed to compare the performance of a feature-based and a contextual embedding-based model. In Tables 3 and 4, the QWKs of the feature- and the embedding-based DNN predictions on the test data can be found in the (prompt-specific) third and fourth rows, respectively. For comparison, the same results measures in PCC can be found in the Appendix (Tables 10 and 11). Across traits and prompts, the feature-based model outperformed the embedding-based model in 11 out of 16 cases. However, the performance of both models was similar. The feature-based model achieved an overall average QWK of $\:{\stackrel{-}{\text{Q}\text{W}\text{K}}}_{features}=\:0.614$ ($\:\stackrel{-}{\text{P}\text{C}\text{C}}=0.673$), and the embedding-based model achieved an overall average of $\:{\stackrel{-}{\text{Q}\text{W}\text{K}}}_{embeddings}=\:0.563$ ($\:\stackrel{-}{\text{P}\text{C}\text{C}}=0.625$). The T-test across traits and prompts implied no significant differences between the two approaches (*p* = .345). In addition, it became apparent that the embedding-based model fell short, especially in the trait *organization* of the two MEWS prompts (QWK differences of $\:{{\Delta\:}\text{Q}\text{W}\text{K}}_{MEWS\:1}=0.31$ and $\:{{\Delta\:}\text{Q}\text{W}\text{K}}_{MEWS\:2}=0.33$, respectively), while performing almost equally well across all other traits (and prompts). Furthermore, the same pattern for the trait *organization* was evident in ASAP 2 but not in ASAP 1. Nevertheless, this finding seems plausible as (even contextual) embeddings might not carry information about an essay’s (meta-) structure, which is relevant for human annotators judging student essays. In contrast, such information, for instance the number of paragraphs, is represented in the feature set.

**Table 3:** Quadratic Weighted Kappa Across ASAP Essay Traits

	Content	Organization	Word choice	Sentence fluency	Conventions
ASAP 1					
N-Gram reg.	0.536	0.511	0.515	0.491	0.481
Feature reg.	0.678	0.635	0.672	0.636	0.623
Feature DNN	0.693	0.657	**0.690**	0.645	0.639
DistilBERT	0.713	0.666	0.677	0.675	**0.666**
Hybrid	.**743**	.**672**	0.673	.**681**	0.648
M. & B. (2018)^1^	0.67	0.60	0.64	0.62	0.61
M. & B. (2020)^2^	0.703	0.664	0.675	0.648	0.638
ASAP 2					
N-Gram reg.	0.552	0.541	0.548	0.396	0.402
Feature reg.	0.637	0.658	0.686	0.672	0.684
Feature DNN	0.664	0.662	0.698	0.688	**0.699**
DistilBERT	0.651	0.591	0.686	0.674	0.685
Hybrid	**0.688**	.**686**	.**715**	.**736**	0.685
M. & B. (2018)^1^	0.61	0.58	0.60	0.59	0.62
M. & B. (2020)^2^	0.617	0.623	0.630	0.603	0.601

The best performing model for each trait and prompt is printed in bold. reg. = ridge regression

^1^Performance benchmarks in terms of QWK from Mathias and Bhattacharyya (2018)

^2^Performance benchmarks in terms of QWK from Mathias and Bhattacharyya (2020)

**Table 4:** Model Performances Across MEWS Essay Traits

	Content	Organization	Language quality
MEWS 1 (AD)			
N-Gram reg.	0.330	0.142	0.442
Feature reg.	0.423	0.509	0.662
Feature DNN	0.380	0.482	0.648
DistilBERT	0.396	0.171	0.556
Hybrid	**0.463**	.**521**	**0.698**
Human Threshold^1^	0.66	0.68	0.71
MEWS 2 (TE)			
N-Gram reg.	0.289	0.167	0.464
Feature reg.	**0.435**	0.507	0.654
Feature DNN	0.377	0.517	0.688
DistilBERT	0.355	0.192	0.667
Hybrid	0.376	**0.528**	**0.723**
Human threshold^1^	0.52	0.77	0.72

The best performing model for each trait and prompt is printed in bold. reg. = ridge regression

^1^Human rater agreement in terms of QWK

Beside these differences in the trait *organization*, no systematic superiority of one approach was found across traits. For ASAP, QWKs even implied more systematic differences between prompts than between traits. While the embedding-based DNN outperforms the feature-based DNN in four out of five traits of ASAP 1, the feature-based DNN outperforms the embedding-based DNN in all traits of ASAP 2.

Furthermore, we compared these two models against two simpler baseline models. These baseline models used a ridge regression with n-grams versus the feature input. The prompt-specific first and second rows of Tables 3 and 4 Model represent the test data performance for each trait. The comparison of our DNN target models with the n-gram baseline model revealed that both target models consistently outperformed the baseline. The one-sided T-tests indicated significant performance advantages ($\:{p}_{features}$ < 0.001 and $\:{p}_{embeddings}$ = 0.010, respectively). However, comparisons to the feature-based linear regression baseline only partly revealed advantages for the target models, and T-tests were not significant ($\:{p}_{features}=0.818$, $\:\:{p}_{embeddings}=0.465$). The feature-based linear baseline model even performed consistently above the embedding-based DNN across all traits of the MEWS prompts ($\:{\stackrel{-}{{\Delta\:}\text{Q}\text{W}\text{K}}}_{embeddings\:vs.\:\:baseline\:1}=-0.01$). The feature-based DNN also fell short in four out of six traits in the MEWS prompts compared to the feature baseline ($\:{\stackrel{-}{{\Delta\:}\text{Q}\text{W}\text{K}}}_{features\:vs.\:\:baseline\:1}=-0.02$). Regarding the two ASAP prompts, however, the two DNN approaches almost consistently performed above the feature-based baseline. However, the differences were small ($\:{\stackrel{-}{{\Delta\:}\text{Q}\text{W}\text{K}}}_{features\:vs.\:\:baseline\:2}=0.02$ and $\:{\stackrel{-}{{\Delta\:}\text{Q}\text{W}\text{K}}}_{embeddings\:vs.\:\:baseline\:2}=0.01$). These relatively small advantages imply that nonlinearities and interactions among features (as well as embeddings) were of minor importance when scoring the essay traits (see also Table 9 in the Appendix). This finding also matches expectations as raters typically follow strict judgment guidelines for benchmark scoring. Such guidelines are almost exclusively based on linear, additive scoring rules.

### Hybrid Architecture

The goal of RQ 2 was to compare a hybrid model architecture containing both feature types – linguistic features and contextual embeddings – to the single-resource models. The trait-specific test set performance of the hybrid model is represented in the fifth row of each prompt in Tables 3 and 4. The hybrid model achieved an average performance of $\:{\stackrel{-}{\text{Q}\text{W}\text{K}}}_{hybrid}=\:0.640$ ($\:\stackrel{-}{\text{P}\text{C}\text{C}}=0.681$). As expected, the hybrid model outperformed the single-resource models in most traits (12 out of 16) across prompts. However, the one-sided T-test comparing the performance of the feature-based model to the hybrid was not significant ($\:{\stackrel{-}{{\Delta\:}\text{Q}\text{W}\text{K}}}_{hybrid-features}=0.03$, $\:p=\:0.507$). The difference between the embedding-based DNN and the hybrid also failed significance ($\:{\stackrel{-}{{\Delta\:}\text{Q}\text{W}\text{K}}}_{hybrid-embeddings}=0.08$, $\:p=\:0.156$). Despite the non-significant results, the hybrid consistently proved to perform better than the single-resource models across prompts and traits. This finding meets expectations and is in line with recent findings from holistic scoring (Bai & Stede, 2022; Uto et al., 2020). Furthermore, it implies that both types of input indeed capture partially different text information relevant for scoring essay traits. Thus, both types of input complemented each other to a certain extent, even when most of the text information relevant for assessing essay traits seemed to be captured by both input types. This is plausible when considering that both single- resource models already achieved high QWK in almost all traits and prompts.

A closer look at the different traits revealed that the largest average gains comparing the feature model to the hybrid were apparent in the *content* and *language* traits ($\:{\stackrel{-}{{\Delta\:}\text{Q}\text{W}\text{K}}}_{content}=\:0.04,\:\:{\stackrel{-}{{\Delta\:}\text{Q}\text{W}\text{K}}}_{language}=\:0.04)$. However, the advantages of the hybrid model were only slightly smaller for the *organization* traits on average ($\:{\stackrel{-}{{\Delta\:}\text{Q}\text{W}\text{K}}}_{organization}=\:0.02$). For this comparison, the three language traits w*ord choice*,* sentence fluency*, and *conventions*, used in the ASAP + + analytic scoring rubric, were all used as measures for *language* (to match the less detailed dimensionality of the MEWS rubric). However, a closer look at these three language traits in ASAP + + revealed that the performance on the trait *conventions* was least likely to benefit from the combined input of the hybrid model.

These findings are not surprising as the employed features hardly capture content-related information, and the contextual embeddings were a decisive contribution in this respect. Therefore, the most considerable performance gains had been expected for trait *content*. However, the powerful properties of contextual embeddings regarding language and writing style have also been repeatedly proven in recent years. In this context, the successful interplay of features and contextual embeddings for the *language* traits also seems to be expectable.

A closer look at the trait-specific gains comparing the embeddings-based and the hybrid model revealed the highest performance gains for the *organization* traits ($\:{\stackrel{-}{{\Delta\:}\text{Q}\text{W}\text{K}}}_{organization}=\:0.16$, $\:{\stackrel{-}{{\Delta\:}\text{Q}\text{W}\text{K}}}_{content}=\:0.02,\:\:{\stackrel{-}{{\Delta\:}\text{Q}\text{W}\text{K}}}_{language}=\:0.02$). As mentioned above, this result also corresponds to our expectations, since embeddings hardly capture any information about the meta-structure of essays.

### Addition and Ablation Tests

**Fig. 4:**
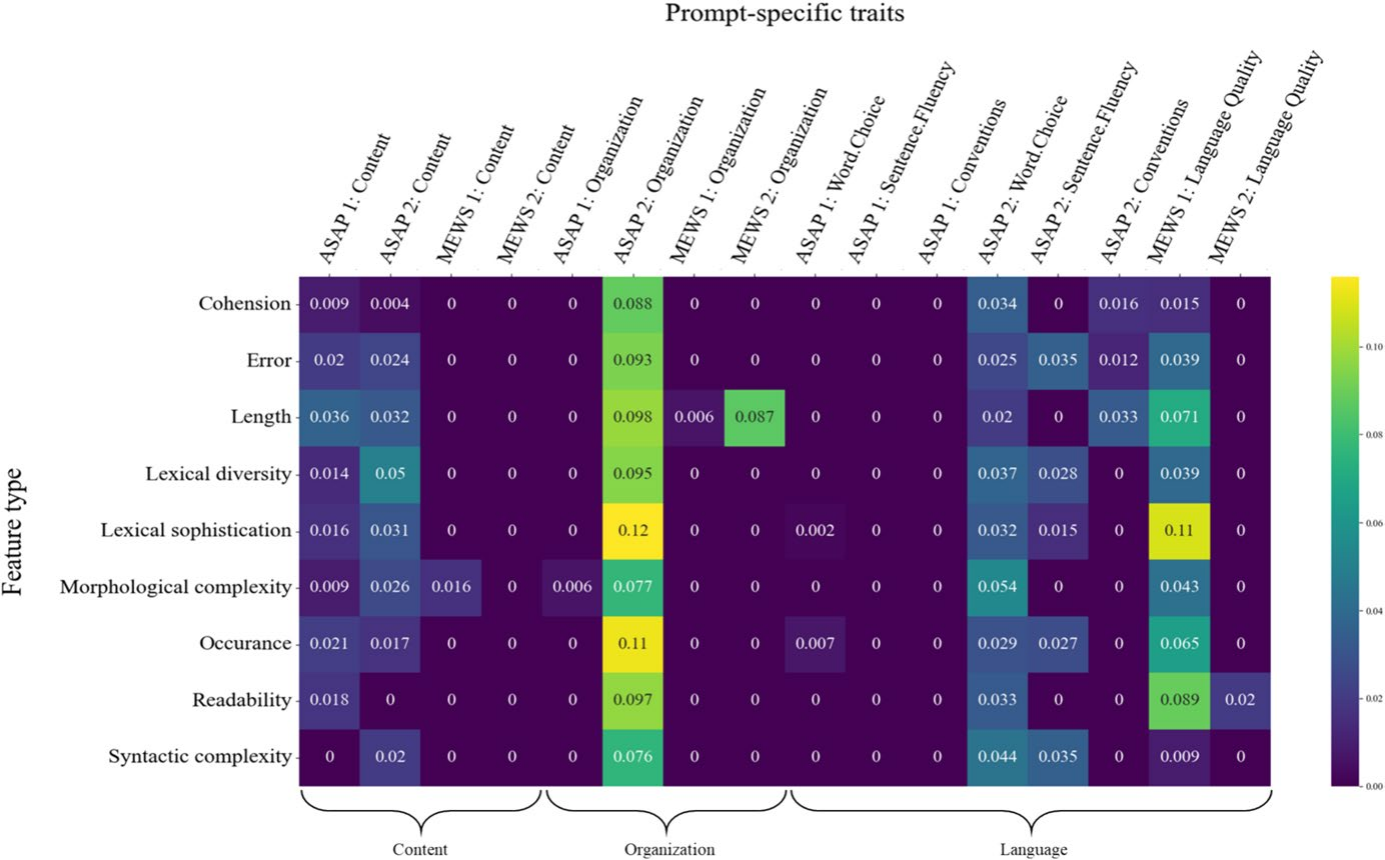
Addition Tests Tracking Highest Performance Gains ($\:\varDelta\:QWK$) by Adding one Type of Features to the Embedding-Based Models

 To shed more light on the interplay of contextual embeddings and specific feature types when scoring certain essay traits, we ran two series of tests. In the first series, we iteratively supplemented the embedding-based DNN with one feature type (addition tests). In doing so, we tracked the performance gains of these extended models compared to the DNNs that only relied on embeddings. These comparisons allowed us to explore essay characteristics that could hardly be covered by the contextual embeddings but by the appropriate features, thus improving model performance. Figure 4 presents the respective results in terms of QWK change (i.e., $\:{\Delta\:}\text{Q}\text{W}\text{K}$) for each trait and prompt. Across prompts, performance gains on the *content* traits appeared most often when the morphological complexity features supplemented the contextual embeddings. Supplementing the contextual embedding input, length features turned out to be most important for the *organization* traits. Furthermore, lexical sophistication, error, and occurrence features were most likely to achieve performance advantages across the *language* traits. Again, these findings seem reasonable. Length features describing the meta-structure of the essays provide structural information that embeddings cannot capture. In the context of the assessment of *language* traits, text characteristics, such as spelling or grammar errors, are also no natural ingredients of embeddings but are undoubtedly important to judge the language quality of student essays. The same applies to lexical sophistication and occurrence features, which describe aspects of language quality inaccessible by contextual embeddings. An interesting finding is that morphological complexity features were most relevant for the *content* traits. On the one hand, morphological complexity might not carry content-related information. On the other hand, comparatives and superlatives might be highly relevant inflections in argumentative writing. For example, these inflections can be relevant when different arguments are contrasted or weighted to draw conclusions. Students’ ability to contrast and weigh is essential for good argumentative writing.

**Fig. 5:**
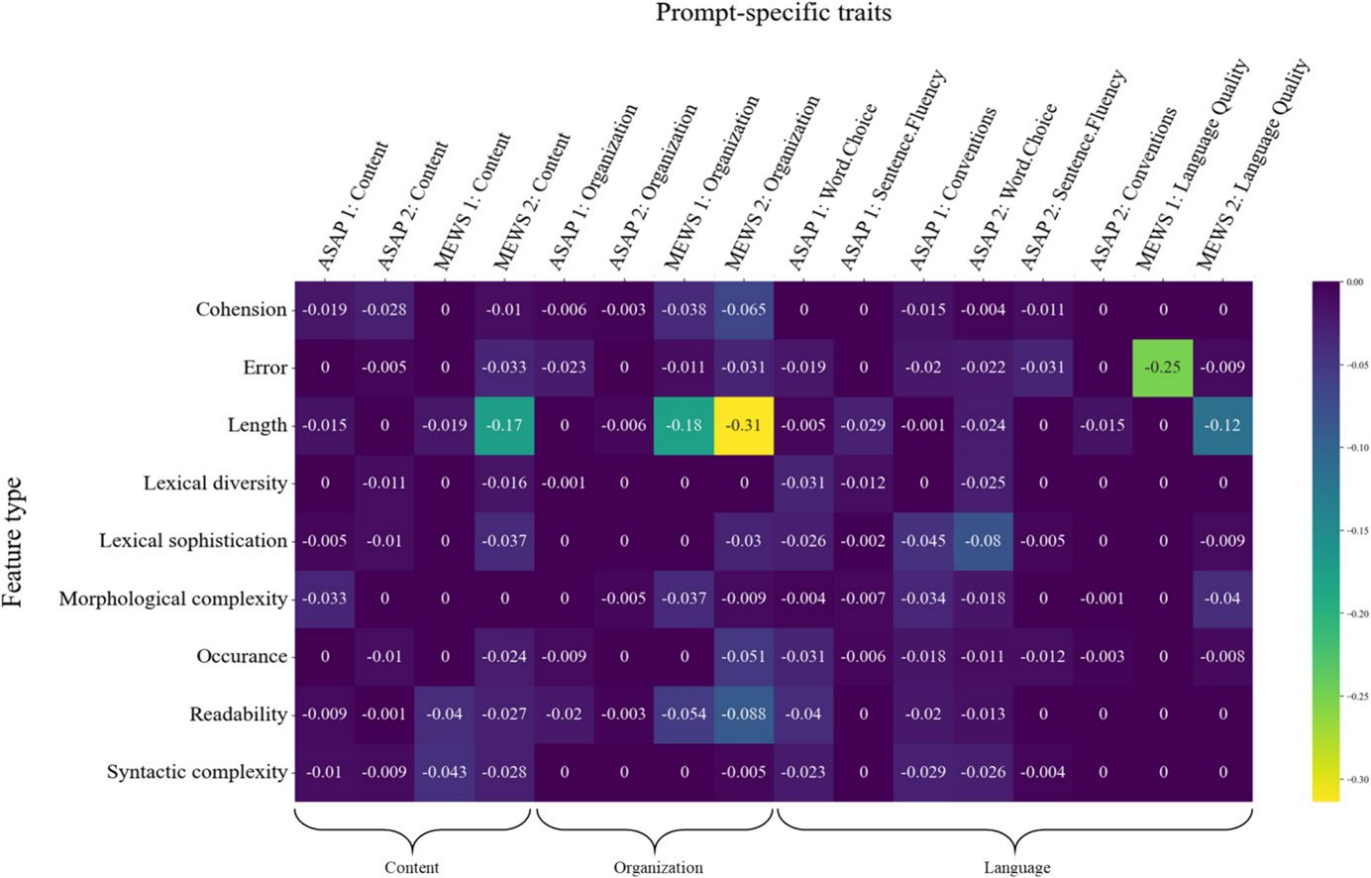
Ablation Tests Tracking Highest Performance Drops ($\:\varDelta\:QWK$) by Removing one Type of Features from the Hybrid Models

In the second series of tests, we explored the unique contribution of single feature types. We used the complete hybrid architecture and iteratively removed one of the nine feature types (ablation tests). Figure 5 shows the performance drops for each trait- and prompt-specific model and the nine re-analyses. Consistent performance drops across prompts indicate that a particular feature type contains trait-relevant information and that the contextual embeddings and the other features do not capture this information. The results imply that the performance of models for the *content* traits dropped across all four prompts when readability and syntactic complexity features were removed. Therefore, both seem to contain unique information relevant to the assessment of content that the other feature types or contextual embeddings could not capture. Consistent performance drops were apparent when removing cohesion and, again, readability features from the trait models for *organization*. When removing occurrence, length, or error features, performance almost consistently decreased across the *language* traits. Throughout traits, length features, in particular, emerged as an essential feature type capturing important and unique text characteristics for judging the student essays.

### Cross-Prompt Scoring

Tables 5 and 6 present the cross-prompt performance of the DNN models trained on the ASAP and MEWS corpora respectively. The analyses show that across models and traits, the performance drop ($\:\varDelta\:\text{Q}\text{W}\text{K}$) when comparing the within-prompt performance to the cross-prompt performance was between − 0.01 and − 0.30 ($\:\varDelta\:\text{P}\text{C}\text{C}\:\text{r}\text{a}\text{n}\text{g}\text{e}=[-0.15;-0.01]$). For the test data of the MEWS 2 *organization* trait, the embedding-based model trained on MEWS 1 even slightly outperformed the embedding-based model trained on MEWS 2 ($\:\varDelta\:\text{Q}\text{W}\text{K}=0.02;$ i.e., the cross-prompt performance was better than the within-prompt performance). However, the embedding-based models generally worked very poorly for the MEWS organization trait.

Regarding the models trained on the MEWS prompts and ASAP 1, the feature-based models outperformed the embedding-based models in cross-prompt performance across traits. However, the embedding-based models trained on ASAP 2 consistently outperformed the feature-based model in cross-prompt performance. T-tests revealed no significant cross-prompt scoring advantages for the feature-based DNN ($\:{\stackrel{-}{\text{Q}\text{W}\text{K}}}_{features}=\:0.49$; $\:{\stackrel{-}{\text{P}\text{C}\text{C}}}_{features}=\:0.52$) compared to the embedding-based model ($\:{\stackrel{-}{\text{Q}\text{W}\text{K}}}_{embeddings}=\:0.42;\:{\stackrel{-}{\text{P}\text{C}\text{C}}}_{embeddings}=\:0.45$) (*p* = .131). Unsurprisingly, the results of these cross-prompt performance comparisons are in line with the within-prompt patterns (see Tables 3 and 4). However, adjusting for the within-prompt performance still implies slight advantages for the feature approach ($\:{\stackrel{-}{\varDelta\:\text{Q}\text{W}\text{K}}}_{features}=\:-0.12$, $\:{\stackrel{-}{\varDelta\:\text{Q}\text{W}\text{K}}}_{embeddings}=\:-0.15$; $\:{\stackrel{-}{\varDelta\:\text{P}\text{C}\text{C}}}_{features}=\:-0.09$, $\:{\stackrel{-}{\varDelta\:\text{P}\text{C}\text{C}}}_{embeddings}=\:-0.11$).

**Table 5:** ASAP Cross-Prompt Scoring Performance in Terms of QWK (and Comparing Cross-Prompt and Within-Prompt Performance)

	Content		Organization		Word choice		Sentence fluency		Conventions	
	Test: ASAP 1	Test: ASAP 2	Test: ASAP 1	Test: ASAP 2	Test: ASAP 1	Test: ASAP 2	Test: ASAP 1	Test: ASAP 2	Test: ASAP 1	Test: ASAP 2
Training: ASAP 1										
Features	0.69	0.56 (-0.13)	0.66	0.52 (-0.14)	0.69	0.55 (-0.14)	0.65	0.56 (-0.09)	0.64	0.54 (-0.10)
DistilBERT	0.71	0.60 (-0.11)	0.67	.**56** (-0.09)	0.68	.**58** (-0.10)	0.68	0.62 (-0.16)	0.67	**0.56** (-0.11)
Hybrid	0.74	.**61** (-0.13)	0.67	0.50 (-0.17)	0.67	0.51 (-0.16)	0.68	**0.63** (-0.05)	0.65	**0.56** (-0.09)
	Test: ASAP 2	Test: ASAP 1	Test: ASAP 2	Test: ASAP 1	Test: ASAP 2	Test: ASAP 1	Test: ASAP 2	Test: ASAP 1	Test: ASAP 2	Test: ASAP 1
Training: ASAP 2										
Features	0.66	**0.54** (-0.12)	0.66	**0.51** (-0.15)	0.70	**0.54** (-0.16)	0.69	**0.54** (-0.15)	0.70	0.50 (-0.20)
DistilBERT	0.65	0.43 (-0.22)	0.59	0.36 (-0.23)	0.69	0.46 (-0.23)	0.67	0.50 (-0.27)	0.69	0.49 (-0.20)
Hybrid	0.69	0.43 (-0.26)	0.69	0.45 (-0.24)	0.72	0.48 (-0.24)	0.74	0.51 (-0.23)	0.69	.**51** (-0.18)

Differences between cross-prompt and within-prompt performance are represented in brackets ($\:\varDelta\:$QWK)

**Table 6:** MEWS Cross-Prompt Scoring Performance in Terms of QWK (and Comparing Cross-Prompt and Within-Prompt Performance)

	Content		Organization		Language quality	
	Test: MEWS 1	Test: MEWS 2	Test: MEWS 1	Test: MEWS 2	Test: MEWS 1	Test: MEWS 2
Training: MEWS 1						
Features	0.38	**0.22** (-0.16)	0.48	0.40 (-0.08)	0.65	0.56 (-0.09)
DistilBERT	0.40	0.12 (-0.28)	0.17	0.16 (-0.01)	0.56	0.47 (-0.09)
Hybrid	0.46	0.16 (-0.30)	0.52	**0.43** (-0.09)	0.70	**0.59** (-0.11)
	Test: MEWS 2	Test: MEWS 1	Test: MEWS 2	Test: MEWS 1	Test: MEWS 2	Test: MEWS 1
Training: MEWS 2						
Features	0.38	.**33** (-0.05)	0.52	**0.49** (-0.03)	0.69	0.62 (-0.07)
DistilBERT	0.36	0.18 (-0.18)	0.19	0.21 (0.02)	0.67	0.54 (-0.13)
Hybrid	0.38	0.30 (-0.08)	0.53	0.46 (-0.07)	0.72	.**66** (-0.06)

Differences between cross-prompt and within-prompt performance are represented in brackets ($\:\varDelta\:$QWK)

The hybrid model also outperformed the embeddings-based approach regarding cross-prompt scoring but also just fell short of the feature-based model on average ($\:{\stackrel{-}{\text{Q}\text{W}\text{K}}}_{hybrid}=\:0.48;\:{\stackrel{-}{\text{P}\text{C}\text{C}}}_{hybrid}=\:0.52$). Surprisingly, adjusting for the within-prompt performance, the hybrid model even performed worse than both single approaches ($\:{\stackrel{-}{\varDelta\:\text{Q}\text{W}\text{K}}}_{hybrid}=\:-0.16$, $\:{\stackrel{-}{\varDelta\:\text{P}\text{C}\text{C}}}_{hybrid}=\:-0.12$). However, T-tests revealed that these differences were not statistically significantly different from zero.

Furthermore, we also explored trait-specific cross-prompt performance losses. Across models, the most remarkable drop in model performance from within-prompt to cross-prompt scoring was revealed for the *content* traits ($\:{\stackrel{-}{\varDelta\:\text{Q}\text{W}\text{K}}}_{content}=\:-0.17$; $\:{\stackrel{-}{\varDelta\:\text{P}\text{C}\text{C}}}_{content}=-0.09$). The comparably smallest drop was apparent for the *organization* traits ($\:{\stackrel{-}{\varDelta\:\text{Q}\text{W}\text{K}}}_{language}=-0.10$; $\:\:{\stackrel{-}{\varDelta\:\text{P}\text{C}\text{C}}}_{language}=-0.05$). This result is also in line with expectations, as the topics of the individual prompts differ and the features’ importance can therefore vary depending on the prompt. In addition, indicators for language and organizational text quality might be more stable across different writing prompts.

## Discussion

In the present study, we compared different supervised ML models for automated trait scoring of student essays using four argumentative prompts from L1 and L2 upper secondary students. Results implied small performance advantages for trait-specific models based on an extensive set of features compared to models based on contextual embeddings that stem from the pre-trained transformer DistilBERT. The differences between the two approaches were particularly evident in the organization traits. However, since contextual embeddings do not require extensive feature engineering, this approach can serve as a valuable baseline model for essay trait scoring, performing significantly better than an n-gram baseline model in our experiments. The hybrid approach, using both input types, consistently outperformed the two single resource models across traits. Addition tests revealed that the performance of the embedding-based models was consistently enhanced in content assessment when combined with morphological complexity features. In addition, performance gains were consistently achieved in organization assessment when combined with length features and in the assessment of language traits when combined with lexical complexity, error, and occurrence features. The feature-based models exhibited slight advantages in cross-prompt scoring over the embedding-based and hybrid models. When comparing trait-specific cross-prompt and within-prompt performance, losses were slightly larger in trait content across ML approaches and prompts compared to organization and language traits.

### Limitations and Future Research

Despite the various models considered and the extensive experiments run, the present study also has limitations that imply several directions for future research.

First, even considering L1 and L2 learners’ essays, the present investigation is limited to upper high school / secondary school students from three countries (American L1 students and German and Swiss L2 students). The performance of different models might vary with learner populations and should be extended, for instance, to primary school (e.g., Trüb et al., under review) or higher education contexts (e.g., Beseiso et al., 2021).

Second, pooling contextualized embeddings on the essay level indeed implies a loss of information that is captured by transformer models. This essay-level pooling approach is only one possibility of using transformer models in AES tasks (see, e.g., Xue et al., 2021). Future studies might explore transformer models’ potential, for example, for feature engineering. Valuable strategies might be to use section-level embeddings or cosine similarities with prompts or best-practice solutions (see Bexte et al., 2022, 2023). Furthermore, sentence-level embeddings can be used for calculating cohesion measures.

Third, in our experiments, we relied on contextual embeddings as fixed features provided by DistilBERT. However, although requiring much more computational costs, transformers can also be fine-tuned to specific tasks. While performance gains were small at best in holistic AES tasks (see, e.g., Mayfield & Black, 2020; Rodriguez et al., 2019), less is known about fine-tuning of transformers for analytic scoring^11^. Future studies could deepen this aspect of transformer models in automated analytic essay scoring.

Fourth, there are other essential topics in AES applications such as fairness and algorithms’ vulnerability to cheating behavior. Future studies could compare feature-based and embedding-based AES models regarding fairness (Schaller et al., 2024) and cheating behavior in trait assessment (see, e.g., Ding et al., 2020; also Bai & Stede, 2022).

Fifth, performance of supervised ML models highly depends on the number of training examples. This might explain to a certain extent the performance differences between ASAP and MEWS prompts in our experiments. However, further systematic experiments varying the amount of training data across ML approaches and prompts would be needed to quantify the relevance of training data size. Such investigations might also consider active learning approaches to minimize the required number of training examples (e.g., Firoozi et al., 2023; Horbach & Palmer, 2016).

Finally, the power of large language models (LLMs; i.e., extensively pre-trained generative transformer models such as GPT-4) have recently entered the AI world. They also offer new possibilities to the field of AES applications. First approaches have, for instance, explored their potential to be included in an LLM-based hybrid model (Mizumoto & Eguchi, 2023).

### Practical Implications

The present study has several implications, especially for creating feedback tools and tutoring systems in the context of student essay evaluation. In our experiments, the feature engineering approach performed as well or better than the embedding approach across essay traits. Since the feature approach can provide more explainability and, thus, more concrete practical information for student feedback, we consider the feature approach as the most promising alley for implementing real-life AES tools. However, in AES applications, an embedding-based DNN approach can serve as a valuable baseline that is easy to set up as no feature engineering is required. Furthermore, our experiments imply that a hybrid approach can increase performance compared to single-resource models. Feature engineering approaches can benefit from embedding-based model inputs, especially scoring content and language quality traits.

In future applications, the hybrid approaches could be chosen for the summative assessment of essay traits if a sufficiently large amount of training data is available. The feature engineering approach, on the other hand, could be used primarily for formative feedback due to its explainability.

## Data Availability

Data (MEWS) and analysis code are provided on OSF (https://doi.org/10.17605/OSF.IO/ZBMXH). In addition, we also reanalyzed the ASAP++ data (https://lwsam.github.io/ASAP++/lrec2018.html).

## Appendix

**Table 7:** Interrater Agreement of the TrACE Analytic Annotation of the MEWS Corpus

	Interrater correlation Mean	Interrater correlation Median	Weighted Cohen’s Kappa Mean	Weighted Cohen’s Kappa Median
Language quality	0.75	0.77	0.72	0.74
MEWS 1 (AD)	0.73	0.73	0.71	0.71
MEWS 2 (TE)	0.75	0.76	0.72	0.73
Organization	0.72	0.73	0.72	0.73
MEWS 1 (AD)	0.68	0.70	0.68	0.70
MEWS 2 (TE)	0.76	0.76	0.77	0.76
Content	0.64	0.64	0.61	0.61
MEWS 1 (AD)	0.68	0.70	0.66	0.67
MEWS 2 (TE)	0.56	0.57	0.52	0.52

**Table 8:** Feature Types

Feature type	Features
Length features	1. Nb. of words 2. Nb. of unique tokens 3. Nb. of letters 4. Nb. of sentences 5. Nb. of paragraphs 6. Nb. of syllables
Occurrence features	1. Nb. of nouns 2. Nb. of verbs 3. Nb. of adjectives 4. Nb. of conjunctions 5. Nb. of adverbs 6. Nb. of possessive pronouns 7. Nb. of unique nouns 8. Nb. of unique verbs 9. Nb. of unique adjectives 10. Nb. of unique adverbs 11. Nb. of “wh”-adverbs 12. Nb. of determiners 13. Nb. of lexical words 14. Nb. of unique lexical words 15. Nb. of foreign words 16. Nb. of stopwords 17. Nb. of formal words 18. Nb. of deictic words 19. Nb. of symbols 20. Nb. of punctuations
Error features	1. Nb. of errors 2. Nb. of grammar errors 3. Nb. of punctuation errors 4. Nb. of typos errors 5. Ratio Nb. of errors / words 6. Ratio Nb. of grammar errors / words 7. Ratio Nb. of punctuation errors / words 8. Ratio Nb. of typos errors / words
Morphological complexity	1. Nb. of comparatives 2. Nb. of superlatives 3. Nb. of finite verbs 4. Nb. of non-third person singular verb 5. Nb. of infinitive verbs 6. Ratio of comparatives 7. Ratio of superlatives 8. Ratio of finite verbs 9. Ratio of non-third person singular verb 10. Ratio of infinitive verbs
Cohesion	1. Nb. of connectors 2. Nb. of unique connectors 3. Mean noun overlap with previous sentence 4. Mean verb overlap with previous sentence 5. SD noun overlap with previous sentence 6. SD verb overlap with previous sentence 7. Ratio of connectors 8. Ratio of unique connectors
Readability	1. Flesch Score 2. Dale-Chall Score 3. Gunning-Flog Index 4. Integration Cost 5. Average nb. of sentences per 100 words 6. Average nb. of words per 100 letters 7. Words per sentences 8. Type-token ratio easy words 9. Type-token ratio easy nouns 10. Type-token ratio easy verbs 11. Type-token ratio easy adverbs 12. Type-token ratio easy adjectives 13. Integration cost 14. Heylinghen-F-Score
Lexical diversity	1. Type-token ratio 2. Type-token ratio nouns 3. Type-token ratio verbs 4. Type-token ratio adjectives 5. Type-token ratio conjunctions 6. Type-token ratio lexical words 7. Type-token ratio functional words 8. Type-token ratio deictic words 9. Type-token ratio “wh”-adverbs 10. Type-token ratio infinitive verbs 11. Global edit distance
Lexical sophistication	1. BNC easy words 2. NGSL easy words 3. SUBLEX easy words 4. BNC easy nouns 5. NGSL easy nouns 6. SUBLEX easy nouns 7. BNC easy verbs 8. NGSL easy verbs 9. SUBLEX easy verbs 10. Brown Frequencies token 11. Brown Frequencies type 12. Brown Frequencies lex. words 13. Brown Frequencies func. words 14. Thorndike Frequencies token 15. Thorndike Frequencies type 16. Thorndike Frequencies lex. words 17. Thorndike Frequencies func. words 18. MRC Frequencies token 19. MRC Frequencies type 20. MRC Frequencies lex. words 21. MRC Frequencies func. words
Syntactic complexity	1. Nb. subordinate clauses 2. Nb. fragment sentences 3. Nb. of noun phrases 4. Mean tokens before main verb 5. Nb. of complex noun phrases 6. Nb. of unknown constituents 7. Nb. of postnominal modifiers per complex noun phrase 8. Integration cost 9. Ratio subordinate clauses 10. Ratio fragment sentences 11. Ratio of noun phrases 12. SD tokens before main verb 13. Ratio of complex noun phrases 14. Ratio of unknown constituents 15. Ratio of postnominal modifiers per complex noun phrase

We additionally calculated several ratios and distribution parameters (i.e., means and standard deviations) for some of the features

**Table 9:** Best Hyperparameter Settings for Each Model, Trait, and Dataset

	Trait	Learning rate	Number of Layers	Number of Units	Dropout Rate
ASAP 1					
Features	Content	0.001	1	128	0.3
	Organization	0.005	1	64	0.4
	Word Choice	0.0005	1	256	0.4
	Sentence Fluency	0.001	2	64	0.2
	Conventions	0.001	1	256	0.3
DistilBERT	Content	0.001	0	128	0.3
	Organization	0.001	0	256	0.4
	Word Choice	0.001	0	128	0.4
	Sentence Fluency	0.001	0	128	0.4
	Conventions	0.001	0	128	0.4
Hybrid	Content	0.001	1	128	0.3/0.4
	Organization	0.005	1	64	0.4/0.4
	Word Choice	0.0005	1	256	0.4/0.4
	Sentence Fluency	0.001	2	64	0.2/0.4
	Conventions	0.001	1	256	0.3/0.4
ASAP 2					
Features	Content	0.001	1	128	0.3
	Organization	0.0005	2	128	0.2
	Word Choice	0.001	2	256	0.3
	Sentence Fluency	0.0005	2	256	0.3
	Conventions	0.0005	2	256	0.4
DistilBERT	Content	0.001	0	256	0.4
	Organization	0.0005	2	256	0.3
	Word Choice	0.001	0	64	0.4
	Sentence Fluency	0.001	0	64	0.4
	Conventions	0.001	0	256	0.4
Hybrid	Content	0.001	1	128	0.3/0.4
	Organization	0.0005	2	128	0.2/0.4
	Word Choice	0.001	2	256	0.3/0.4
	Sentence Fluency	0.0005	2	256	0.3/0.4
	Conventions	0.0005	2	256	0.4/0.4
MEWS 1 (AD)					
Features	Content	0.0005	1	128	0.4
	Organization	0.0005	1	256	0.3
	Language	0.0005	2	128	0.3
DistilBERT	Content	0.0005	2	64	0.4
	Organization	0.005	2	64	0.4
	Language	0.005	2	256	0.2
Hybrid	Content	0.001	1	128	0.4/0.7
	Organization	0.001	1	256	0.2/0.7
	Language	0.001	1	64	0.3/0.4
MEWS 2 (TE)					
Features	Content	0.001	1	256	0.2
	Organization	0.0005	2	256	0.3
	Language	0.0005	2	128	0.3
DistilBERT	Content	0.005	2	64	0.3
	Organization	0.001	2	256	0.3
	Language	0.001	0	256	0.2
Hybrid	Content	0.001	1	256	0.2/0.7
	Organization	0.001	2	256	0.3/0.7
	Language	0.001	2	128	0.3/0.5

**Table 10:** Pearson Correlation Coefficients for ASAP within- and Cross-Prompt Performance

	Content		Organization		Word Choice		Sentence Fluency		Conventions	
	Test: ASAP 1	Test: ASAP 2	Test: ASAP 1	Test: ASAP 2	Test: ASAP 1	Test: ASAP 2	Test: ASAP 1	Test: ASAP 2	Test: ASAP 1	Test: ASAP 2
Training: ASAP 1										
Features	0.711	0.659 (-0.05)	0.674	0.653 (-0.02)	**0.709**	0.646 (-0.06)	0.665	0.631 (-0.04)	0.667	0.640 (-0.03)
DistilBERT	0.722	0.634 (-0.09)	0.678	0.644 (-0.04)	0.690	0.655 (-0.03)	0.686	0.654 (-0.04)	0.677	0.643 (-0.04)
Hybrid	**0.730**	0.654 (-0.08)	**0.679**	0.612 (-0.07)	0.682	0.624 (-0.06)	**0.702**	0.665 (-0.03)	**0.681**	0.664 (-0.02)
	Test: ASAP 2	Test: ASAP 1	Test: ASAP 2	Test: ASAP 1	Test: ASAP 2	Test: ASAP 1	Test: ASAP 2	Test: ASAP 1	Test: ASAP 2	Test: ASAP 1
Training: ASAP 2										
Features	**0.688**	0.688 (-0.00)	**0.687**	0.658 (-0.02)	**0.719**	0.644 (-0.08)	**0.707**	0.644 (-0.07)	**0.722**	0.620 (-0.10)
DistilBERT	0.671	0.658 (-0.01)	0.638	0.599 (-0.04)	0.703	0.639 (-0.06)	0.692	0.636 (-0.05)	0.705	0.613 (-0.10)
Hybrid	0.667	0.666 (-0.00)	0.679	0.648 (-0.03)	0.712	0.649 (-0.06)	0.704	0.641 (-0.06)	0.706	0.632 (-0.08)

Differences between cross-prompt and within-prompt performance are represented in brackets ($\:\varDelta\:$PCC)

**Table 11:** Pearson Correlation Coefficients for MEWS Within- and Cross-Prompt Performance

	Content		Organization		Language quality	
	Test: MEWS 1	Test: MEWS 2	Test: MEWS 1	Test: MEWS 2	Test: MEWS 1	Test: MEWS 2
Training: MEWS 1						
Features	0.440	0.318 (-0.12)	0.556	0.495 (-0.06)	0.680	0.671 (-0.01)
DistilBERT	0.490	0.260 (-0.23)	0.267	0.243 (-0.03)	0.605	0.589 (-0.02)
Hybrid	**0.510**	0.381 (-0.23)	**0.593**	0.559 (-0.03)	**0.728**	0.708 (-0.02)
	Test: MEWS 2	Test: MEWS 1	Test: MEWS 2	Test: MEWS 1	Test: MEWS 2	Test: MEWS 1
Training: MEWS 2						
Features	**0.432**	0.388 (-0.04)	0.579	0.569 (-0.01)	0.718	0.651 (-0.07)
DistilBERT	0.368	0.257 (-0.11)	0.289	0.251 (-0.04)	0.694	0.580 (-0.11)
Hybrid	0.420	0.360 (-0.06)	**0.594**	0.559 (-0.03)	**0.746**	0.690 (-0.06)

Differences between cross-prompt and within-prompt performance are represented in brackets ($\:\varDelta\:$PCC)

**Table 12:** Test Set Performance of the Fixed versus Fine-Tuned DistilBERT Version

	Content		Organization		Word Choice		Sentence Fluency		Conventions	
	Fixed	Fine-tuned	Fixed	Fine-tuned	Fixed	Fine-tuned	Fixed	Fine-tuned	Fixed	Fine-tuned
ASAP 1	0.713	0.647	0.666	0.544	0.677	0.608	0.675	0.643	0.666	0.660
ASAP2	0.651	0.573	0.591	0.584	0.686	0.625	0.674	0.653	0.685	0.626
	Content		Organization		Language					
	Fixed	Fine-tuned	Fixed	Fine-tuned	Fixed	Fine-tuned				
MEWS 1	0.396	0.363	0.171	0.210	0.556	0.585				
MEWS 2	0.355	0.332	0.192	0.294	0.667	0.610				

The fine-tuning process included cross-validation with the same data splits as used in the main article. We kept all DistilBERT layers except the last layer frozen. This resulted in around 7 million trainable neurons (ca. 11%) in the DistilBERT architecture. During training, we tried several learning rate setting

## Footnotes

1 https://www.kaggle.com/c/asap-aes.

2 https://languagetool.org.

3 https://osf.io/zbmxh/.

4 https://osf.io/zbmxh/.

5 https://spacy.io.

6 https://languagetool.org.

7 It should be noted that there is also a validation set of around 600 examples per prompt provided by ASAP. However, this validation set was neither used by Taghipour and Ng for holistic AES nor by Mathias & Bhattacharyya for trait AES. To ensure comparability of findings, we aligned our experiments with these authors’ procedures.

8 https://osf.io/zbmxh/.

9 A DNN with zero Dense layers corresponds to a standard linear regression. However, we added a dropout mechanism in our application (Fig. 3).

10 https://scikit-learn.org/stable/.

11 Encouraged by a reviewer’s comment, we provide a first comparison between our fixed contextual embedding approach and a fine-tuning approach in Appendix Table A6.

## References

[CR1] Alikaniotis, D., Yannakoudakis, H., & Rei, M. (2016). Automatic text scoring using neural networks. In K. Erk & N. A. Smith (Eds.), *Proceedings of the 54th Annual Meeting of the Association for Computational Linguistics, ACL 2016* (pp. 715–725). Association for Computational Linguistics. 10.18653/v1/P16-1068

[CR2] Andrade, H. L. (2018). Feedback in the context of self-assessment. In A. A. Lipnevich & J. K. Smith (Eds.), *The Cambridge handbook of instructional feedback* (pp. 376–408). Cambridge University Press. 10.1017/9781316832134.019

[CR3] Attali, Y., & Powers, D. (2008). A developmental writing scale. *ETS Research Report Series*, *2008*(1). 10.1002/j.2333-8504.2008.tb02105.x

[CR4] Bai, X., & Stede, M. (2022). A survey of current machine learning approaches to Student Free-text evaluation for intelligent tutoring. *International Journal of Artificial Intelligence in Education,**33*(4), 1–39. 10.1007/s40593-022-00323-0 10.1007/s40593-022-00323-0 PMC9707071 36467629

[CR5] Bergstra, J., & Bengio, Y. (2012). Random search for hyper-parameter optimization. *Journal of Machine Learning Research,**13*(2), 281–305.

[CR6] Beseiso, M., & Alzahrani, S. (2020). An empirical analysis of BERT Embedding for Automated Essay Scoring. *International Journal of Advanced Computer Science and Applications*, *11*(10). 10.14569/IJACSA.2020.0111027

[CR7] Beseiso, M., Alzubi, O. A., & Rashaideh, H. (2021). A novel automated essay scoring approach for reliable higher educational assessments. *Journal of Computing in Higher Education,**33*(3), 727–746. 10.1007/s12528-021-09283-1

[CR8] Bexte, M., Horbach, A., & Zesch, T. (2022). Similarity-Based Content Scoring - How to Make S-BERT Keep Up With BERT. In E. Kochmar, J. C. Burstein, A. Horbach, R. Laarmann-Quante, N. Madnani, A. Tack, V. Yaneva, Z. Yuan, & T. Zesch (Eds.), *Proceedings of the 17th Workshop on Innovative Use of NLP for Building Educational Applications (BEA 2022)* (pp. 118–123). Association for Computational Linguistics. 10.18653/v1/2022.bea-1.16

[CR9] Bexte, M., Horbach, A., & Zesch, T. (2023). Similarity-Based Content Scoring - A more Classroom-Suitable Alternative to Instance-Based Scoring? In A. Rogers, J. Boyd-Graber, & N. Okazaki (Eds.), *Findings of the Association for Computational Linguistics: ACL 2023* (pp. 1892–1903). Association for Computational Linguistics. 10.18653/v1/2023.findings-acl.119

[CR10] Brezina, V., & Pallotti, G. (2019). Morphological complexity in written L2 texts. *Second Language Research, 35*(1), 99–119. 10.1177/0267658316643125

[CR11] Chassab, R. H., Zakaria, L. Q., & Tiun, S. (2021). Automatic essay Scoring: A review on the feature analysis techniques. *International Journal of Advanced Computer Science and Applications*, *12*(10). 10.14569/IJACSA.2021.0121028

[CR12] Chen, X., & Meurers, D. (2016). *CTAP: A Web-Based Tool Supporting Automatic Complexity Analysis.*10.17863/CAM.39630

[CR13] Chen, J., Fife, J. H., Bejar, I. I., & Rupp, A. A. (2016). Building e-rater ^®^ scoring models using machine learning methods. *ETS Research Report Series,**2016*(1), 1–12. 10.1002/ets2.12094

[CR14] Cohen, J. (1968). Weighted kappa: Nominal scale agreement with provision for scaled disagreement or partial credit. *Psychological Bulletin,**70*(4), 213–220. 10.1037/h0026256 19673146 10.1037/h0026256

[CR15] Condon, W., & Elliot, N. (2022). Liz Hamp Lyons: a life in writing assessment. *Assessing Writing,**53*, 100651. 10.1016/j.asw.2022.100651

[CR16] Crossley, S. A. (2019). Using human judgments to examine the validity of automated grammar, syntax, and mechanical errors in writing. *Journal of Writing Research,**11*(2), 251–270. 10.17239/jowr-2019.11.02.01

[CR17] Crossley, S. A. (2020). Linguistic features in writing quality and development: An overview. *Journal of Writing Research,**11*(3), 415–443. 10.17239/jowr-2020.11.03.01

[CR18] Crossley, S. A., & Holmes, L. (2023). Assessing receptive vocabulary using state–of–the–art natural language processing techniques. *Journal of Second Language Studies,**6*(1), 1–28. 10.1075/jsls.22006.cro

[CR19] Crossley, S. A., & McNamara, D. S. (2014). Does writing development equal writing quality? A computational investigation of syntactic complexity in L2 learners. *Journal of Second Language Writing,**26*, 66–79. 10.1016/j.jslw.2014.09.006

[CR20] Crossley, S. A., Kyle, K., & McNamara, D. S. (2016). The development and use of cohesive devices in L2 writing and their relations to judgments of essay quality. *Journal of Second Language Writing,**32*, 1–16. 10.1016/j.jslw.2016.01.003

[CR21] Crossley, S. A., Kyle, K., & McNamara, D. S. (2017). Sentiment analysis and social cognition engine (SEANCE): An automatic tool for sentiment, social cognition, and social-order analysis. *Behavior Research Methods,**49*(3), 803–821. 10.3758/s13428-016-0743-z 27193159 10.3758/s13428-016-0743-z

[CR22] Crowhurst, M. (1983). Syntactic complexity and writing quality: A review. *Canadian Journal of Education / Revue Canadienne De L’éducation,**8*(1), 1. 10.2307/1494403

[CR23] Dasgupta, T., Naskar, A., Dey, L., & Rupsa, S. (2018). Augmenting textual qualitative features in deep convolution recurrent neural network for automatic essay scoring. *Proceedings of the 5th Workshop on Natural Language Processing Techniques for Educational Applications*, 93–102. 10.18653/v1/W18-3713

[CR24] Deane, P., Yan, D., Castellano, K., Attali, Y., Lamar, M., Zhang, M., Blood, I., Bruno, J. V., Li, C., [Chen], Cui, W., Ruan, C., Appel, C., James, K., Long, R., & Qureshi, F. (2024). Modeling writing traits in a formative essay Corpus. ETS Research Report Series, Article ets2.12377. 10.1002/ets2.12377. Advance online publication

[CR25] Ding, Y., Riordan, B., Horbach, A., Cahill, A., & Zesch, T. (2020). Don’t take nswvtnvakgxpm for an answer –The surprising vulnerability of automatic content scoring systems to adversarial input. In D. Scott, N. Bel, & C. Zong (Eds.), *Proceedings of the 28th International Conference on Computational Linguistics* (pp. 882–892). International Committee on Computational Linguistics. 10.18653/v1/2020.coling-main.76

[CR26] Doewes, A., Kurdhi, N., & Saxena, A. (2023). Evaluating quadratic weighted kappa as the standard performance metric for automated essay scoring. In *16th International Conference on Educational Data Mining, EDM 2023* (pp. 103–113). International Educational Data Mining Society (IEDMS).

[CR27] Firoozi, T., Mohammadi, H., & Gierl, M. J. (2023). Using active learning methods to strategically select essays for automated scoring. *Educational Measurement: Issues and Practice,**42*(1), 34–43. 10.1111/emip.12537

[CR28] Fleckenstein, J., Keller, S., Krüger, M., Tannenbaum, R. J., & Köller, O. (2020). Linking TOEFL iBT^®^ writing rubrics to CEFR levels: Cut scores and validity evidence from a standard setting study. *Assessing Writing,**43*, 100420. 10.1016/j.asw.2019.100420

[CR29] Fleckenstein, J., Meyer, J., Jansen, T., Keller, S., & Köller, O. (2020). Is a long essay always a good essay? The effect of text length on writing Assessment. *Frontiers in Psychology,**11*, 562462. 10.3389/fpsyg.2020.562462 33071888 10.3389/fpsyg.2020.562462 PMC7544919

[CR30] Gamon, M., Chodorow, M., Leacock, C., & Tetreault, J. (2013). Grammatical error detection in Automatic Essay Scoring and Feedback. In M. D. Shermis & J. C. Burstein (Eds.), *Handbook on automated essay evaluation: Current applications and new directions* (pp. 251–266). Routledge Academic.

[CR31] Horbach, A., & Palmer, A. (2016). Investigating Active Learning for Short-Answer Scoring. In J. Tetreault, J. C. Burstein, C. Leacock, & H. Yannakoudakis (Eds.), *Proceedings of the 11th Workshop on Innovative Use of NLP for Building Educational Applications* (pp. 301–311). Association for Computational Linguistics. 10.18653/v1/W16-0535

[CR32] Horbach, A., Scholten-Akoun, D., Ding, Y., & Zesch, T. (2017). Fine-grained essay scoring of a complex writing task for native speakers. In J. Tetreault, J. Burstein, C. Leacock, & H. Yannakoudakis (Eds.), *Proceedings of the 12th Workshop on Innovative Use of NLP for Building Educational Applications* (pp. 357–366). Association for Computational Linguistics. 10.18653/v1/W17-5040

[CR33] Hussein, M. A., Hassan, H., & Nassef, M. (2019). Automated language essay scoring systems: A literature review. *PeerJ Computer Science,**5*, e208. 10.7717/peerj-cs.208 10.7717/peerj-cs.208 PMC7924549 33816861

[CR34] Injadat, M., Moubayed, A., Nassif, A. B., & Shami, A. (2021). Machine learning towards intelligent systems: Applications, challenges, and opportunities. *Artificial Intelligence Review,**54*(5), 3299–3348. 10.1007/s10462-020-09948-w

[CR35] Jarvis, S. (2013). Capturing the diversity in lexical diversity. *Language Learning,**63*(s1), 87–106. 10.1111/j.1467-9922.2012.00739.x

[CR36] Ke, Z., & Ng, V. (2019). Automated essay scoring: A survey of the state of the art. In T. Eiter & S. Kraus (Eds.), *Proceedings of the Twenty-Eighth International Joint Conference on Artificial Intelligence* (pp. 6300–6308). International Joint Conferences on Artificial Intelligence Organization. 10.24963/ijcai.2019/879

[CR37] Keller, S. D., Fleckenstein, J., Krüger, M., Köller, O., & Rupp, A. A. (2020). English writing skills of students in upper secondary education: Results from an empirical study in Switzerland and Germany. *Journal of Second Language Writing,**48*, 100700. 10.1016/j.jslw.2019.100700

[CR38] Keller, S. D., Lohmann, J., Trüb, R., Fleckenstein, J., Meyer, J., Jansen, T., & Möller, J. (2024). Language quality, content, structure: What analytic ratings tell us about EFL writing skills at upper secondary school level in Germany and Switzerland. *Journal of Second Language Writing,**65*, 101129. 10.1016/j.jslw.2024.101129

[CR39] Kumar, V. S., & Boulanger, D. (2021). Automated essay scoring and the deep learning black box: How are rubric scores determined? *International Journal of Artificial Intelligence in Education,**31*(3), 538–584. 10.1007/s40593-020-00211-5

[CR40] Kusuma, J. S., Halim, K., Pranoto, E. J. P., Kanigoro, B., & Irwansyah, E. (2022). Automated Essay Scoring Using Machine Learning. In *2022 4th International Conference on Cybernetics and Intelligent System (ICORIS)* (pp. 1–5). IEEE. 10.1109/ICORIS56080.2022.10031338

[CR41] Kyle, K., Crossley, S. A., & Berger, C. (2018). The tool for the automatic analysis of lexical sophistication (TAALES): Version 2.0. *Behavior Research Methods,**50*(3), 1030–1046. 10.3758/s13428-017-0924-4 28699123 10.3758/s13428-017-0924-4

[CR42] Lagakis, P., & Demetriadis, S. (2021). Automated essay scoring: A review of the field. In *2021 International Conference on Computer, Information and Telecommunication Systems (CITS)* (pp. 1–6). IEEE. 10.1109/CITS52676.2021.9618476

[CR43] Lample, G., & Conneau, A. (2019). *Cross-lingual Language Model Pretraining.*10.48550/arXiv.1901.07291

[CR44] Lewis, M., Liu, Y., [Yinhan], Goyal, N., Ghazvininejad, M., Mohamed, A., Levy, O., Stoyanov, V., & Zettlemoyer, L. (2019). *BART: Denoising Sequence-to-Sequence Pre-training for Natural Language Generation, Translation, and Comprehension.*10.48550/arXiv.1910.13461

[CR45] Linacre, J. M. (1994). *Many-facet rasch measurement* (2nd ed.). Mesa Press.

[CR46] Linacre, J. M. (2019). *Facets (Version 3.82.1)*. [Computer software].

[CR47] Mathias, S., & Bhattacharyya, P. (2018). ASAP++: Enriching the ASAP automated essay grading dataset with essay attribute scores. *Proceedings of the Eleventh International Conference on Language Resources and Evaluation (LREC 2018)*. https://aclanthology.org/L18-1187. Accessed 10.12.2023.

[CR48] Mathias, S., & Bhattacharyya, P. (2020). Can Neural Networks Automatically Score Essay Traits? In J. C. Burstein, E. Kochmar, C. Leacock, N. Madnani, I. Pilán, H. Yannakoudakis, & T. Zesch (Eds.), *Proceedings of the Fifteenth Workshop on Innovative Use of NLP for Building Educational Applications* (pp. 85–91). Association for Computational Linguistics. 10.18653/v1/2020.bea-1.8

[CR49] Mayfield, E., & Black, A. W. (2020). Should You Fine-Tune BERT for Automated Essay Scoring? In J. C. Burstein, E. Kochmar, C. Leacock, N. Madnani, I. Pilán, H. Yannakoudakis, & T. Zesch (Eds.), *Proceedings of the Fifteenth Workshop on Innovative Use of NLP for Building Educational Applications* (pp. 151–162). Association for Computational Linguistics. 10.18653/v1/2020.bea-1.15

[CR50] McNamara, D. S., Graesser, A. C., McCarthy, P. M., & Cai, Z. (2014). *Automated evaluation of text and discourse with Coh-Metrix*. Cambridge University Press. 10.1017/CBO9780511894664

[CR51] Mesgar, M., & Strube, M. (2018). A Neural Local Coherence Model for Text Quality Assessment. In E. Riloff, D. Chiang, J. Hockenmaier, & J. Tsujii (Eds.), *Proceedings of the 2018 Conference on Empirical Methods in Natural Language Processing* (pp. 4328–4339). Association for Computational Linguistics. 10.18653/v1/D18-1464

[CR52] Mitkov, R., & Voutilainen, A. (2012). *Part-of-Speech Tagging* (Vol. 1). Oxford University Press. 10.1093/oxfordhb/9780199276349.013.0011

[CR53] Mizumoto, A., & Eguchi, M. (2023). Exploring the potential of using an AI language model for automated essay scoring. *Research Methods in Applied Linguistics,**2*(2), 100050. 10.1016/j.rmal.2023.100050

[CR54] Nadeem, F., Nguyen, H., Liu, Y., [Yang], & Ostendorf, M. (2019). Automated Essay Scoring with Discourse-Aware Neural Models. In H. Yannakoudakis, E. Kochmar, C. Leacock, N. Madnani, I. Pilán, & T. Zesch (Eds.), *Proceedings of the Fourteenth Workshop on Innovative Use of NLP for Building Educational Applications* (pp. 484–493). Association for Computational Linguistics. 10.18653/v1/W19-4450

[CR55] Nivre, J. (2010). Dependency parsing. *Language and Linguistics Compass,**4*(3), 138–152. 10.1111/j.1749-818X.2010.00187.x

[CR56] Perelman, L. (2014). When the state of the art is counting words. *Assessing Writing,**21*, 104–111. 10.1016/j.asw.2014.05.001

[CR57] Pitler, E., & Nenkova, A. (2008). Revisiting readability: A unified framework for predicting text quality. In M. Lapata & H. T. Ng (Eds.), *Proceedings of the 2008 Conference on Empirical Methods in Natural Language Processing* (pp. 186–195). Association for Computational Linguistics.

[CR58] Ramesh, D., & Sanampudi, S. K. (2022). An automated essay scoring systems: A systematic literature review. *Artificial Intelligence Review,**55*(3), 2495–2527. 10.1007/s10462-021-10068-2 34584325 10.1007/s10462-021-10068-2 PMC8460059

[CR59] Richards, B. (1987). Type/token ratios: What do they really tell us? *Journal of Child Language,**14*(2), 201–209. 10.1017/S0305000900012885 3611238 10.1017/s0305000900012885

[CR60] Robitzsch, A., & Steinfeld, J. (2018). Item response models for human ratings: Item response models for human ratings: Overview, estimation methods, and implementation in R. *Psychological Test and Assessment Modeling,**60*(1), 101–139.

[CR61] Rodriguez, P. U., Jafari, A., & Ormerod, C. M. (2019). *Language models and Automated Essay Scoring.*10.48550/arXiv.1909.09482

[CR62] Rupp, A. A., Casabianca, J. M., Krüger, M., Keller, S., & Köller, O. (2019). Automated essay scoring at scale: A case study in Switzerland and Germany. *ETS Research Report Series,**2019*(1), 1–23. 10.1002/ets2.12249

[CR63] Sanh, V., Debut, L., Chaumond, J., & Wolf, T. (2019). *DistilBERT, a distilled version of BERT: smaller, faster, cheaper and lighter*. https://arxiv.org/pdf/1910.01108v4. Accessed 10.12.2023.

[CR64] Schaller, N. J., Ding, Y., Horbach, A., Meyer, J., & Jansen, T. (2024). Fairness in Automated Essay Scoring: A Fairness in Automated Essay Scoring: A Comparative Analysis of Algorithms on German Learner Essays from Secondary Education. In E. Kochmar, M. Bexte, J. C. Burstein, A. Horbach, R. Laarmann-Quante, A. Tack, V. Yaneva, & Z. Yuan (Eds.), *Proceedings of the 19th Workshop on Innovative Use of NLP for Building Educational Applications (BEA 2024) (pp. 210–221).* (pp. 210–221)

[CR65] Shen, D., Wang, G., Wang, W., Min, M. R., Su, Q., Zhang, Y., Li, C., [Chunyuan], Henao, R., & Carin, L. (2018). *Baseline Needs More Love: On Simple Word-Embedding-Based Models and Associated Pooling Mechanisms.*10.48550/arXiv.1805.09843

[CR66] Shermis, M. D., & Burstein, J. C. (2003). *Automated essay Scoring*. Routledge. 10.4324/9781410606860

[CR67] Taghipour, K., & Ng, H. T. (2016). A Neural Approach to Automated Essay Scoring. In J. Su, K. Duh, & X. Carreras (Eds.), *Proceedings of the 2016 Conference on Empirical Methods in Natural Language Processing* (pp. 1882–1891). Association for Computational Linguistics. 10.18653/v1/d16-1193

[CR68] TensorFlow Developers. (2024). *TensorFlow [Computer software]*. Zenodo.

[CR69] Uto, M. (2021). A review of deep-neural automated essay scoring models. *Behaviormetrika,**48*(2), 459–484. 10.1007/s41237-021-00142-y

[CR70] Uto, M., & Okano, M. (2020). Robust Neural Automated Essay Scoring Using Item Response Theory. In I. I. Bittencourt, M. Cukurova, K. Muldner, R. Luckin, & E. Millán (Eds.), *Lecture Notes in Computer Science. Artificial Intelligence in Education* (Vol. 12163, pp. 549–561). Springer International Publishing. 10.1007/978-3-030-52237-7_44

[CR71] Uto, M., Xie, Y., & Ueno, M. (2020). Neural automated essay scoring incorporating handcrafted features. *Proceedings of the 28th International Conference on Computational Linguistics* (pp. 6077–6088)

[CR72] Vaswani, A., Shazeer, N., Parmar, N., Uszkoreit, J., Jones, L., Gomez, A. N., Kaiser, L., & Polosukhin, I. (2017). *Attention Is all You Need*. 10.48550/arXiv.1706.03762

[CR73] Wang, X., Lee, Y., & Park, J. (2022). *Automated Evaluation for Student Argumentative Writing: A Survey.*10.48550/arXiv.2205.04083

[CR74] Weigle, S. C. (2002). *Assessing writing*. Cambridge University Press. 10.1017/CBO9780511732997

[CR75] Xue, J., Tang, X., & Zheng, L. (2021). A hierarchical BERT-based transfer learning approach for multi-dimensional essay scoring. *Ieee Access : Practical Innovations, Open Solutions,**9*, 125403–125415. 10.1109/ACCESS.2021.3110683

[CR76] Yan, D. (2020). *Handbook of automated scoring: Theory into practice. Chapman and Hall/CRC statistics in the social and behavioral sciences ser*. CRC Press LLC. https://ebookcentral.proquest.com/lib/kxp/detail.action?docID=6124217

[CR77] Yang, Z., Dai, Z., Yang, Y., Carbonell, J., Salakhutdinov, R., & Le, Q. V. (2019). *XLNet: Generalized Autoregressive Pretraining for Language Understanding.*10.48550/arXiv.1906.08237

[CR78] Zesch, T., Wojatzki, M., & Scholten-Akoun, D. (2015). Task-Independent Features for Automated Essay Grading. In J. Tetreault, J. C. Burstein, & C. Leacock (Eds.), *Proceedings of the Tenth Workshop on Innovative Use of NLP for Building Educational Applications* (pp. 224–232). Association for Computational Linguistics. 10.3115/v1/W15-0626

